# Recently Developed Carbohydrate Based Gelators and Their Applications

**DOI:** 10.3390/gels7010024

**Published:** 2021-02-26

**Authors:** Joedian Morris, Jonathan Bietsch, Kristen Bashaw, Guijun Wang

**Affiliations:** Department of Chemistry and Biochemistry, Old Dominion University, Norfolk, VA 23529, USA; jymorris@odu.edu (J.M.); jbietsch@odu.edu (J.B.); kbashaw@odu.edu (K.B.)

**Keywords:** monosaccharides, hydrogelators, organogelators, supramolecular gels, metallogels, hydrogels, carbohydrates, stimuli-responsive, drug delivery

## Abstract

Carbohydrate based low molecular weight gelators have been an intense subject of study over the past decade. The self-assembling systems built from natural products have high significance as biocompatible materials and renewable resources. The versatile structures available from naturally existing monosaccharides have enriched the molecular libraries that can be used for the construction of gelators. The bottom-up strategy in designing low molecular weight gelators (LMWGs) for a variety of applications has been adopted by many researchers. Rational design, along with some serendipitous discoveries, has resulted in multiple classes of molecular gelators. This review covers the literature from 2017–2020 on monosaccharide based gelators, including common hexoses, pentoses, along with some disaccharides and their derivatives. The structure-based design and structure to gelation property relationships are reviewed first, followed by stimuli-responsive gelators. The last section focuses on the applications of the sugar based gelators, including their utilization in environmental remediation, ion sensing, catalysis, drug delivery and 3D-printing. We will also review the available LMWGs and their structure correlations to the desired properties for different applications. This review aims at elucidating the design principles and structural features that are pertinent to various applications and hope to provide certain guidelines for researchers that are working at the interface of chemistry, biochemistry, and materials science.

## 1. Introduction

In general, there are two major types of gel forming materials, polymers and small molecules. In the formation of useful supramolecular structures, the bottom-up approach of using small molecules with built in functionalities is an important method for the creation of functional supramolecular structures. Among many classes of molecular self-assemblies, low molecular weight gelators (LMWGs) have gained great attention over recent years [1,2,3,4]. LMWGs are small molecules, typically molecular weight less than 2000 D, that are able to form reversible gels in various solvents. The resulting gels are termed physical gels or supramolecular gels due to the non-covalent driving forces of gelation. They can form either organogels in organic solvents or hydrogels in water. The formation of these supramolecular gels relies on non-covalent intermolecular forces, including hydrogen bonding, hydrophobic interactions, π-π stacking, and van der Waals interactions. The gelators form self-assembled, typically fibrillar networks, which immobilize organic solvents or aqueous solutions. Low molecular weight hydrogelators (LMHGs) are particularly interesting since they can form hydrogels that lend themselves to biomedical applications. Supramolecular hydrogels are analogous to polymer hydrogels, except with different physical and chemical properties. They also have different thermo stabilities; for instance, typically the molecular gels are unstable at higher temperatures, but stable at lower temperatures. Additionally, since the formation of supramolecular gels is fully reversible, this may lend these gels to many desirable applications that are complementary to existing polymer-based soft materials.

Supramolecular gels have been explored as new materials with applications in many research fields such as in biomedical research for controlled drug delivery [1,2,3,4], enzyme immobilization [5], optical and electronic devices [6,7,8], and in environmental sciences for the removal of pollutants [9,10]. Metallogels formed by small molecules and metal ions have been studied for their applications as smart materials [11,12,13,14]. While many research endeavors are devoted to the biomedical applications of supramolecular gels [15,16,17], there are also several studies on utilizing the self-assembled gels and metal containing gels for catalysis [18,19,20,21]. Due to the interesting implications in supramolecular chemistry, materials science, and biochemistry, a great deal of effort has been dedicated to finding new types of low molecular weight gelators and understanding their properties and applications, as shown in the recent reviews [22,23,24,25,26]. The structures of low molecular weight organogelators (LMOGs) and LMHGs encompass a wide range of different functionalities. Several classes of organic compounds have been found to be useful building blocks in the design and synthesis of LMWGs. These include amino acids, sugars, cholesterols, amides, and ureas. Carbohydrates are readily available naturally abundant renewable resources. The intrinsic chirality and biocompatibility of sugar derived self-assembling systems can lend them to many special applications such as the formation of liquid crystals, the separation of biomolecules, and as controlled release delivery systems.

## 2. Structure and Gelation Properties of Monosaccharide Derivatives

**Figure 1:**
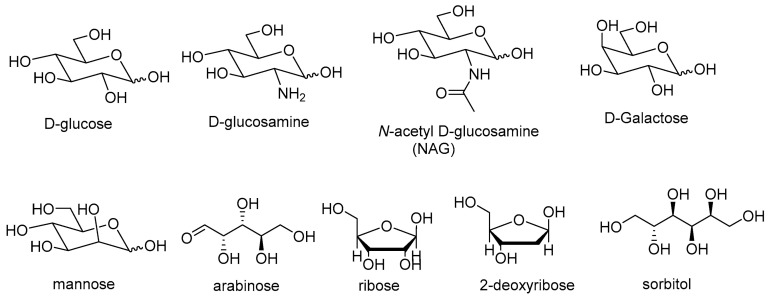
Structures of the sugar starting materials used often for designing low molecular weight gelators (LMWGs).

Carbohydrates are among the important classes of biomolecules, including proteins and nucleic acids. They are also the most abundant renewable resources in nature. They are unique in comparison to other biomolecules in that they contain complex structural diversity, rich stereochemistry, and they play important roles in biology and many disease relevant processes. Sugar-based gelators are likely to be biocompatible and they may find many applications as advanced materials [27,28]. Taking advantage of the natural abundance of monosaccharides and disaccharides, Wang’s group and many others have reported several classes of effective carbohydrate based low molecular weight organogelators and hydrogelators. These include common sugars such as D-glucose, D-glucosamine, N-acetyl-D-glucosamine, D-lactose, and D-maltose as well as sorbitol as shown in Figure 1. Many stimuli-responsive organogelators, which can respond to external stimuli such as light, pH, and enzymes, have also been designed as smart soft materials. In the next several sections, we will briefly discuss gelators derived from the different sugar building blocks starting from D-glucose.

### 2.1. D-Glucose Derivatives as LMWGs

G. Wang and others have been working on the design, synthesis and characterization of sugar-derived LMWGs with an emphasis on establishing the structure and gelation relationships. In the past few years, they have found that several classes of monosaccharide derivatives are effective organogelators and hydrogelators [29,30,31,32,33,34,35,36,37,38,39,40,41,42].

**Figure 2:**
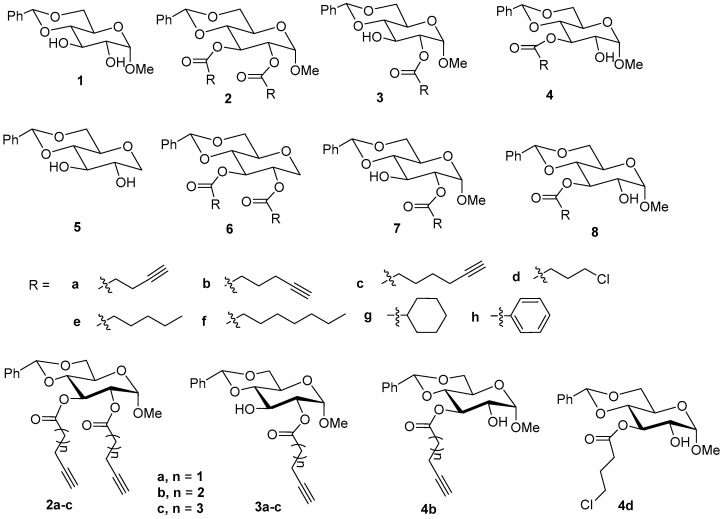
General structures of several classes of glucose ester derivatives as LMWGs [31,32], n represents the number of repeating CH_2_.

Starting from the readily available D-glucose, a variety of LMWGs have been designed and synthesized through the selective functionalization of 4, 6-benzylidene acetal protected α-methyl D-glucose derivatives. Functionalized derivatives of the diol compound **1** at various positions and the resulting gelation properties have been systematically studied. A few libraries of acyl derivatives were synthesized, analyzed and several effective gelators were obtained. As shown in Figure 2, the representative structures include diester derivatives **2** and various monoester derivatives **3** and **4** [32]. Many of the esters with saturated alkyl chains were not good gelators; however, the diester **2h** was able to form a gel in EtOH:H_2_O (*v*/*v* 1:1) at 5 mg/mL and the 3-ester derivative **4d** successfully formed a hydrogel with at 7 mg/mL [32]. The short-chain esters with terminal alkyne functional groups, **2a–c**, **3a–c** and **4a–c** were gelators for several solvents, including hexane and water. This study helped with comprehending the relationship between the structure of the carbohydrate and gelation, which paved the way for the design of novel esters that were more effective gelators.

The influence of the structure at the anomeric position on gelation properties was studied by preparing the 1-deoxy glucose diol headgroup **5** [31]. Similar acyl derivatives, diesters and monoesters **6–8**, were synthesized and analyzed. Diesters **6a**, **6b** and **6h** exhibited gelation in ethanol, aqueous ethanol solutions and aqueous DMSO solutions. These 1-deoxy sugar-derived monoesters showed more solubility in organic solvents than the corresponding 1-methoxy derivatives. Even though the deoxy sugar derivatives with long-chain esters were effective gelators, the presence of the alpha-OMe group was deemed important for molecular self-assembly and gelation properties [31].

**Figure 3:**
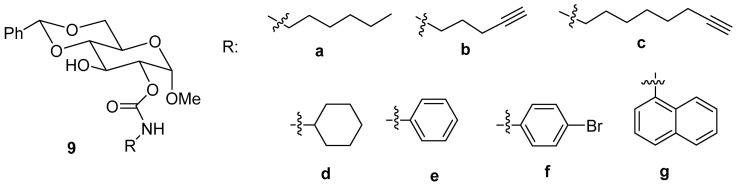
Oxygen linked carbamate derivatives of methyl-a-D-glucopyranoside [34].

Carbohydrate-based molecules with carbamate functional groups have also been found to be effective gelators. This is due to the additional hydrogen bond donating and accepting ability of the carbamate functional group. In comparison to the ester analogs, a wider range of substituents are tolerated and the gels are typically formed at lower concentrations. Several O-linked carbamate derivatives **9** have been synthesized and studied in the Wang group and a few representative compounds are depicted in Figure 3 [34]. The carbamate derivatives using similar alkyl chains are more versatile gelators than their ester counterparts [34]. The alkyl compound **9a** and the cyclohexyl derivative **9d** were effective gelators in 33% aqueous solutions of DMSO or ethanol. In EtOH:H_2_O (*v*/*v* 1:2), **9d** exhibited a very low concentration for gelation at 0.91 mg/mL [34]. The alkynyl carbamates **9b** and **9c**, as well as the aromatic derivatives **9e**–**9f**, formed gels in the DMSO water and ethanol–water mixtures. The naphthyl derivative **9g** formed gels in DMSO water mixture only.

**Figure 4:**
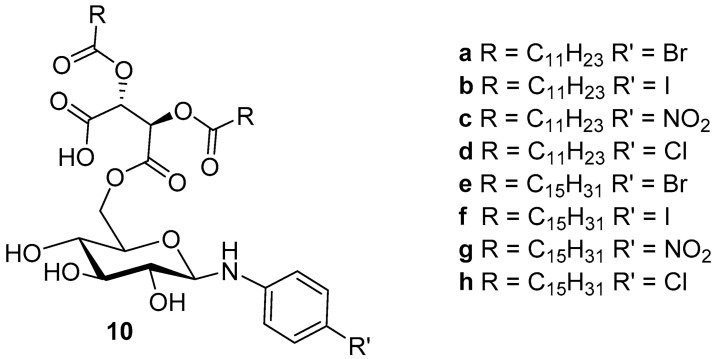
Structures for sugar based glycolipid derivative **10a–h** [43].

Another class of glycolipid derivatives was synthesized and the structures are shown in Figure 4 [43]. Gelation was tested in various aromatic and aliphatic organic solvents. These glycolipids were found to be effective organogelators in mostly aliphatic solvents, with critical gelation concentrations (CGC) of 0.8% (*w*/*v*) for compounds **10b** and **10g**. Compounds **10c**, **10e** and **10h** were effective gelators for several solvents with CGC of 1.0% (*w*/*v*) [43]. Increasing the aliphatic chain in the derivatives does not impact gelation greatly, but changing the R′ group on aniline highly impacts the gelation features. With an eleven carbon chain length in derivatives **a–d**, the best gelation properties were obtained with a nitro and iodo group on the aniline ring [43]. Consequently, with a fifteen carbon chain, the best gelators were obtained with a nitro, chloro and bromo group on the aniline ring, showing that the structure to gelation properties were mostly influenced by the R′ group on the aniline. Overall, the study shows that the alkyl chain length only influenced gelation at the microscopic level, but the substituents on the aniline ring affected the stability and self-assembly of the gels [43].

**Figure 5:**
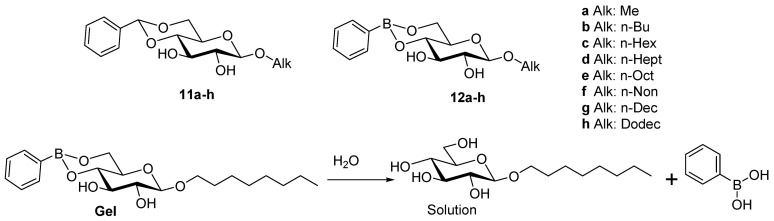
Structures of phenyl boronic ester protected alkyl glucoside derivatives [44].

Ludwig et al. reported a water-sensitive organogelator that is structurally similar to the successful 4,6-benzylidene protected alkyl glucoside derivatives that have been shown to be effective organogelators (Figure 5) [44]. The resulting phenyl boronic protected alkyl glucoside derivatives **12a–h** were found to be effective gelators for toluene, cyclohexane, ethyl myristate and isopropyl palmitate. While the comparable benzylidene protected derivatives are stable in the presence of non-acidic water; the boronate functions are water sensitive. Exposure to water results in hydrolysis to reveal the deprotected glycoside, which are typically not gelators [44]. When exploring the water sensitivity of these organogels, they found that as low as 5% *v*/*v* water to organic solvent solutions were sufficient to cause enough hydrolysis to disrupt the gels [44]. Within 1 h the gels were partially disrupted, and after 3 h the gel state had disappeared. Alternative experiments were carried out replacing the DI water with acetate buffer (pH 4.8) and carbonate buffer (pH 9.8) solutions, yielding similar results [44]. These findings suggest that this sensitivity towards water could make these organogels candidates for applications in topical drug delivery as smart soft materials.

Another phenylboronic acid-related gelator was reported recently by Mahendar et al. [45]. The phenylboronic acid tagged tartaric acid derivative was prepared and it formed a white colored metallogel in the presence of LiOH in DMSO. The gel turned red upon addition of D-(+)-glucose through the reaction with phenyl boronic acid. The glucose containing metallogel also showed increased conductivity [45]. The glucose responsive metallogels could be useful for developing smart materials.

**Figure 6:**
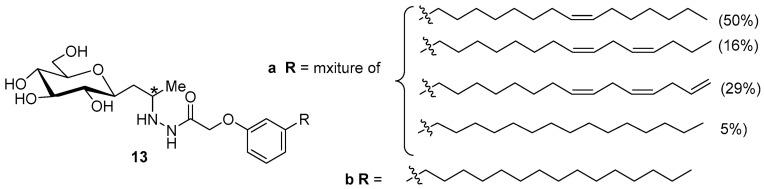
Sophorolipid-based gelators [46].

Sophorolipids are also renewable resources that have been investigated for the development of supramolecular gelators over recent times. Representative structures can be seen in Figure 6 [46]. Compounds **13a–b** were tested in a wide range of organic solvents and water mixtures. These sugar lipids did not form gels in water, nor pure organic solvents; however, they did form gels in mixtures of ethanol, methanol, DMSO and DMF in water at a ratio of 1:5 [46]. The gel with the lowest concentration was compound **13b** in EtOH:H_2_O (*v*/*v* 1:5) at 0.29% *w*/*v* and had a gel-to-sol transition at 48 °C. The gels exhibited twisted densely packed fibrous morphology which were formed via hydrophobic, hydrogen bonding and π-π interactions [46].

**Figure 7:**
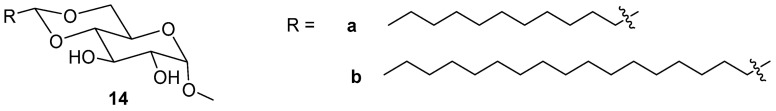
Gelators from methyl alpha-D-glucopyranoside [47].

Ono et al. designed and synthesized a few glucose based LMWGs which formed gels in both aqueous and organic solvents [47]. As shown in Figure 7, these are methyl alpha-D-glucopyranoside derivatives with aliphatic acetal 4,6-protecting groups. Compound **14b**, with an 18-carbon alkyl chain, formed a translucent silicone oil gel that is capable of extrusion. Rheological measurements were used to evaluate the strength of the extruded gel. Frequency sweep curves show a storage modulus (G′) that is consistently larger than the loss modulus (G”), indicating the extruded soft material retained the viscoelastic behavior of a gel. The thixotropic properties of the gel produced from compound **14b** were also investigated in this study. Gels of **14b** in ethanol/water 1:1 and squalene were sheared using a vortex mixer and the gels reformed within 1 h sitting at room temperature undisturbed.

### 2.2. D-Glucosamine and N-Acetyl D-Glucosamine Derivatives

The closely related monosaccharide D-glucosamine and N-acetyl D-glucosamine have been used extensively as the building blocks for the design and synthesis of organogelators and hydrogelators. The presence of the amino group provides an easy point of functionalization and a variety of amide, urea, and carbamate derivatives can be synthesized and studied. Several examples of 4,6 protected derivatives are discussed in the next few sections, followed by the unprotected 2-deoxy-2-amino derivatives.

#### 2.2.1. Glucosamine Derivatives Functionalized at C-2 position from Methyl Glycosides

**Figure 8:**
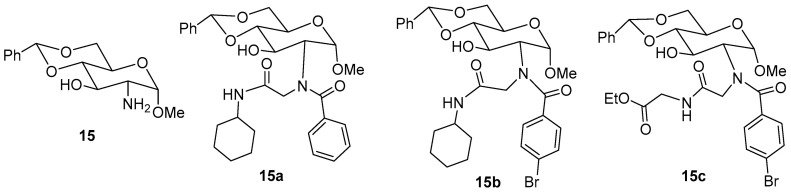
Structure of glucosamine derivative **15** and dipeptoids [37].

A series of dipeptoids were synthesized using multiple component reactions (MCR). The glucosamine derivative **15** was used to prepare several peptoid derivatives **15a–c** as shown in Figure 8 [37]. These compounds showed moderate gelation properties. In comparison to compounds with the free NH groups, the peptoids apparently formed gels through different mechanisms, such as π-π interactions and hydrophobic forces.

**Figure 9:**
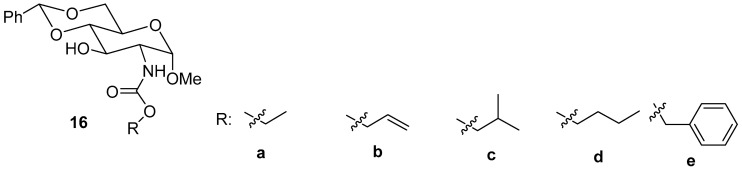
Structures of carbamate derivatives of 4,6-O-benzylidene-methyl-a-D-glucosamine [34].

A series of carbamate derivatives **16** from the glucosamine headgroup **15** was reported (Figure 9) [34]. The N-linked analogs **16a–e** also produced stable gels, with compound **16a** forming a hydrogel at 6.6 mg/mL. The isobutyl derivative **16c** was a versatile gelator for hexane as well as DMSO–water and ethanol–water mixtures. It also formed a hydrogel at 0.4 wt %, but had the lowest gelation concentration in DMSO:H_2_O (*v*/*v* 1:2) at 1.7 mg/mL [34].

**Figure 10:**
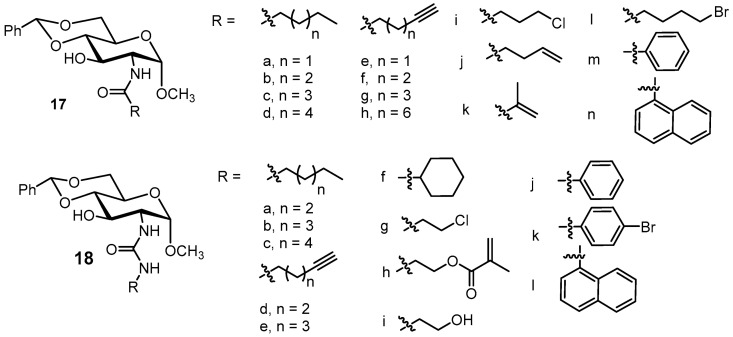
Sugar-based gelators containing amide and urea functions [33], n (in the formula) represents the number of repeating CH_2_.

Amide and urea derivatives have also been synthesized and exhibited gelation properties in a wider panel of solvents making them superior gelators. Urea derivatives, in general, have been studied immensely as gelators and their innate gelation properties are attributed to their ability to form one dimensional hydrogen bonding arrays via the urea functional group which then allows the molecules to assemble into a 3D network [48,49]. Similarly, the amides also form hydrogen bonds which are crucial for their gelation properties [50,51]. Using the headgroup **15**, Goyal et al. have generated a series of amide and urea gelators with varying alkyl and aromatic groups attached [33]. A few representative compounds, **17a–o** and **18a–n**, from their series are shown in Figure 10. The gelation properties of the amides and ureas in water, as well as a variety of organic and aqueous solvents, were studied. Most of the molecules in the series formed gels in 33% aqueous mixtures of ethanol and DMSO, and a few were able to gel ethanol, water and hexane [33]. The hexynyl derivative **17g** was the most efficient forming a stable gel at 0.7 mg/mL in an aqueous solution of ethanol, whilst compound **17m** proved to be a hydrogelator at a concentration of 2 mg/mL [33]. The majority of the amides exhibited excellent gelation properties in aqueous solutions at concentrations less than 5 mg/mL [33]. The heptyl amide **17d** was able to form very stable gels in DMSO and water solutions for up to six months.

Among the ureas synthesized (**18a–l**), the alkyl derivatives showed gel formation in aqueous solutions at low concentrations while the aromatic derivatives formed fewer gels in the selected solvents. The hydroxyethyl urea **18i** was able to form a stable transparent gel in water at 2.2 mg/mL. In general, the gels formed by amides and ureas were more robust compared to the gels from esters and carbamates.

**Figure 11:**
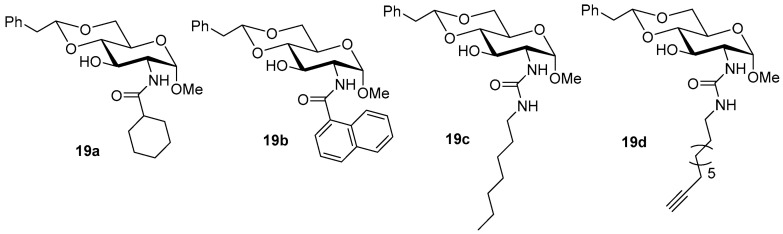
Structures of amide derivatives as organogelators and hydrogelators [42].

With their continuous effort to understand the structural influence on gelation, the Wang research group set out to further evaluate the impact of the 4,6-protective phenyl group by creating a new series of molecules with a methylene group inserted between the sugar and the 4,6-protective site [42]. A few compounds **19a–d** from the series are illustrated in Figure 11. The urea derivatives performed better in the polar organic solvents whilst the amide derivatives were better gelators in aqueous mixtures. A few derivatives formed gels in toluene [42]. The gelation behavior in pump and engine oil was also assessed and the results showed that the aliphatic amide derivatives were effective gelators for these oils [42]. On the other hand, the urea derivatives were not able to form gels in the oils. This may be because the intermolecular attractions among the urea molecules are too strong and hence they were not able to interact with the oil [42]. Compound **19a** formed gels in 6 of the solvents and gave its lowest concentration in pump oil at 2 mg/mL [42]. On the contrary, compound **19b** was only able to form gels in isopropanol and aqueous solutions of DMSO and ethanol [42]. Its lowest concentration of 3.3 mg/mL was seen in EtOH:H**_2_**O (*v*/*v* 1:2) [42]. The urea derivatives **19c** and **19d** were effective gelators in ethylene glycol at 1.6 mg/mL and 3.3 mg/mL, respectively [42].

**Figure 12:**
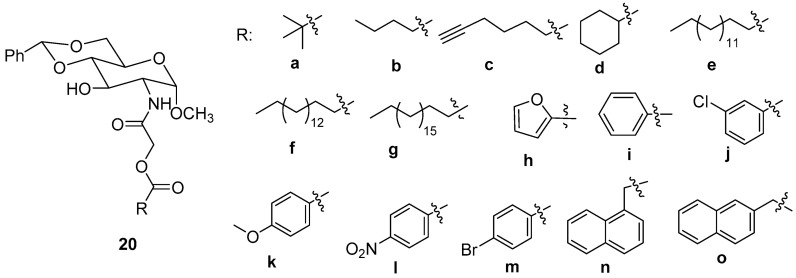
Structures of hybrid ester derivatives from N-acetyl-D-glucosamine [52].

**Figure 13:**
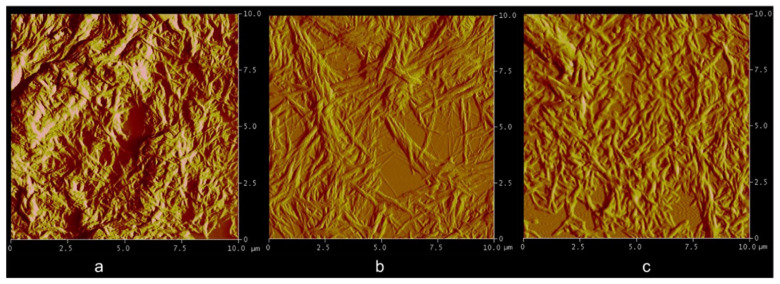
Atomic force microscopy (AFM) phase images of the gels formed by: (**a**) compound **20c** in water at 2.9 mg/mL; (**b**) compound **20j** in DMSO/H_2_O (*v*/*v* 1:2) at 1.4 mg/mL; (**c**) compound **20l** in DMSO/H_2_O (*v*/*v* 1:8) at 2.25 mg/mL with toluidine blue dye (0.031 mg/mL) entrapped. Reprinted with permission from [52]. Copyright 2020 American Chemical Society.

Another series of carbohydrate LMWGs (Figure 12) was developed by further modification of the glucosamine headgroup, using a similar strategy of introducing an ester function to attach to the amide. The hybrid ester compounds formed gels in several of the tested solvents, except the t-butyl ester **20a**. The most versatile gelator, forming gels in ten different solvents, was compound **20b** [52]. The cyclohexyl ester **20d** and the aromatic derivatives are also versatile organogelators, forming gels in many of the tested solvents. The lowest gelation concentrations were 1.4 and 1.3 mg/mL in mixtures of DMSO and water for compounds **20j** and **20k,** respectively. Additionally, gelator **20b** was also able to form a hydrogel at 1.4 mg/mL [52]. The gels were characterized using optical and atomic force microscopy, as well as rheology. These gels exhibited the typical fibrous morphology that is generally seen for gelator self-assembly based on the atomic force microscopy (AFM) images in Figure 13 [52]. Further studies revealed that the gelators were responsive to basic solutions and the lipase enzyme. These gelators also have potential in drug delivery and environmental remediation for dye removal.

#### 2.2.2. Functionalization at C-3 Positions

**Figure 14:**
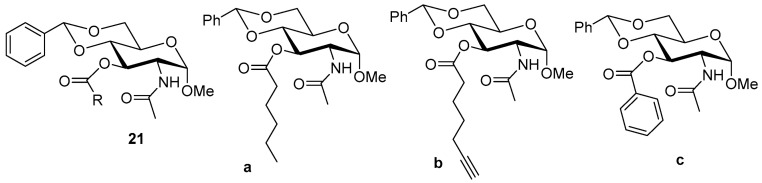
Structures of C-3 ester derivatives of the NAG headgroup [53].

Using the 4,6-benzylidene protected N-acetyl glucosamine as the headgroup, the Wang group reported another series of ester derivatives **21**, synthesized by functionalizing the 3 position of the sugar. As shown in Figure 14, the general 3-O-ester compounds **21** showed promising gelation properties [53]. These molecules were not only efficient gelators, but they were able to solidify a wider variety of solvents. Compound **21a** was a versatile gelator forming gels in pump oil, ethylene glycol, glycerol, as well as mixtures of water with DMSO and ethanol [53]. Compound **21b** also performed well in several solvents, but the aromatic derivatives such as compound **21c** were not multifaceted gelators [53].

**Figure 15:**
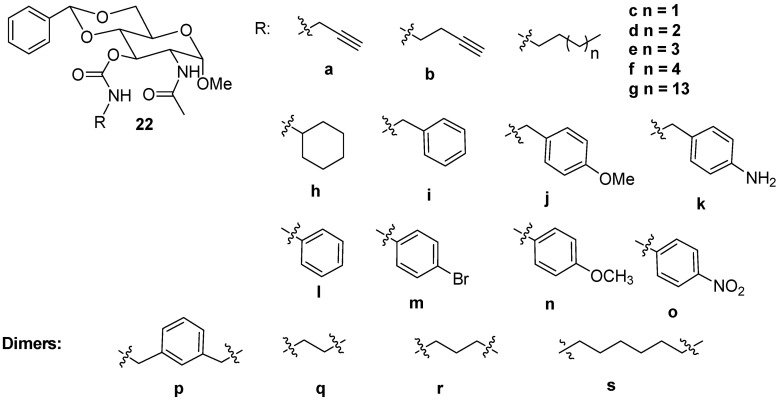
Structures of C-3 carbamates [54], n stands for the number of repeating CH_2_.

**Figure 16:**
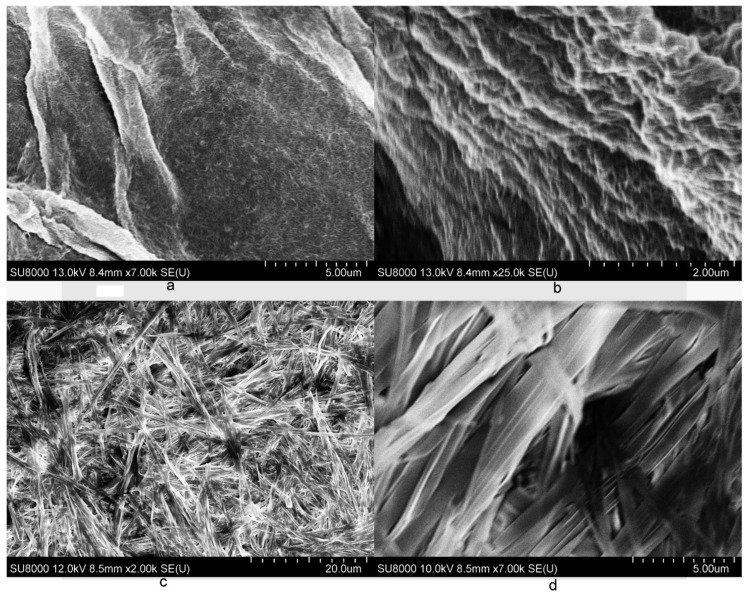
Scanning electron micrographs showing the morphology of two gels: (**a** and **b**) compound **22i** in EtOH:H_2_O (*v*/*v* 1: 1) at 2.5 mg mL^−1^, (**c** and **d**) compound **22j** in EtOH:H_2_O (*v*/*v* 1: 1) at 4.0 mg mL^−1^. Reprinted with permission from [54]. Copyright 2020 The Royal Society of Chemistry under the creative commons license.

Several carbamate functionalized sugars were synthesized from N-acetyl-D-glucosamine and their structures are depicted in Figure 15. Most of the molecules were found to be effective low molecular weight gelators for a wide range of solvents and mixtures. The aliphatic derivatives **22b–g** were able to congeal pump oil at concentrations ranging from 5–20 mg/mL, whilst the aromatic derivatives **22j** and **22l** were superb gelators in DMSO:water (*v*/*v* 1:8) [54]. As evidenced by the scanning electron micrographs in Figure 16, **22i** formed a fibrous network that was very densely packed and **22j** formed crosslinked planar sheets in EtOH:H_2_O (*v*/*v* 1: 1) [54]. Compound **22j** was further tested for its gelation properties in the presence of various metal ions and was found to form gels in the presence of metal ions including Pb^2+^, Cu^2+^, Cu^2+^, Zn^2+^, and Fe^2+^ [54]. Overall, the aliphatic derivatives performed better than most of the aromatic derivatives, and the best performing gelators were compounds **22j** and **22l** [54]. This research adds to the knowledge on how structure affects gelation properties and can be utilized to generate new classes of LMWGs for many applications.

#### 2.2.3. From Unprotected D-Glucosamine

**Figure 17:**
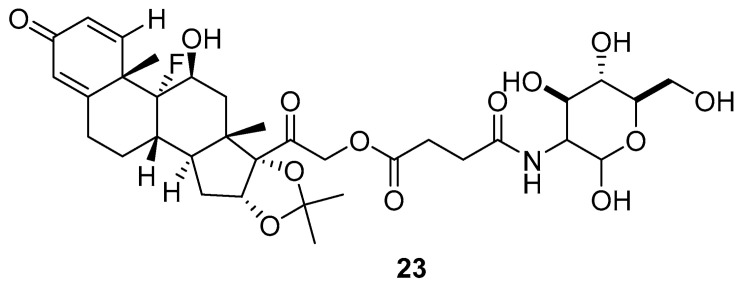
Triamcinolone acetonide-based gelators with a sugar moiety [55].

Steroids have also been investigated widely for their gelation properties. Triamcinolone acetonide (TA) is a common steroidal drug for treating uveitis (eye inflammation); however, it harbors adverse side effects [55]. With this problem in mind, Xiong and his colleagues set out to create a prodrug of the abovementioned steroid that can be injected and possibly reduce the harmful effects. Compound **23,** depicted in Figure 17, was synthesized by attaching glucosamine and succinate to TA [55]. Gelator **23** generated a spontaneous gel in water at a concentration of 0.25 wt % and had thixotropic properties that allowed for it to be injected [55]. Subsequent efficacy and cytotoxicity studies concluded that the hydrogel was able to successfully deliver TA to its active site via injection with comparable efficacy and minimal retinal toxicity when compared to the TA suspension [55]. Overall, their results indicated that the hydrogel prodrug shows promise as another medicinal strategy for treating uveitis [55].

The use of multicomponent gelator systems allows for another way to effectively design gelators through molecular co-assembly. Most articles on these systems have investigated two-component systems, which are divided into three classes: (**1**) A system comprising of the gelator molecule and another component that is not capable of forming a gel; (**2**) A system in which both units are able to form a gel independently and (**3**) the third system consists of two molecules that do not form a gel on their own, but do so when combined [56,57]. The first system is generally designed when there is a need to manage or change the functional properties of the gel. In the second system, the morphology varies based on how they assemble at the molecular level [57]. Self-assembly can result in different types of network; both components can self-sort and give two types of fibers within the gel independently, a mixed self-sorted network is formed, or the components can form alternating assemblies within the fibers [57].

**Figure 18:**
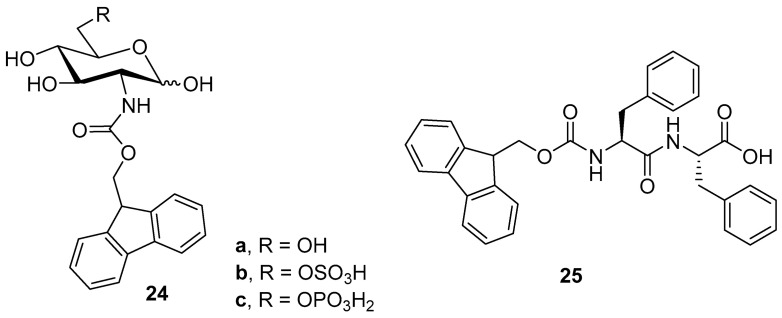
Structures of carbohydrate amphiphiles and a peptide amphiphilic molecule [59,60].

Glucosamine-Fmoc derivatives have been prepared and studied for their gelation properties [58]. Brito et al. reported a two-component gelator system from the precursors in Figure 18 and they were investigated for culturing cells. Due to the fact that compounds **24a** and **25** were soluble at different conditions with reduced solubility in water, compounds **24b** and **24c** were synthesized to improve the solubility of compound **24a** in water [59]. Compound **25** was able to co assemble with **24b** and **24c** at different ratios and the gelation mechanism gave a network that had the carbohydrate units on the outer section and the peptide formed the core-shell of the fibers [59]. Further investigations revealed that the bi-component gel system was not cytotoxic and has the potential to be utilized as a biomimetic for proteoglycans [59]. In a related study, the gelator precursor, N-fluorenylmethyloxycarbonylglucosamine-6-phosphate (compound **24c** in Figure 18) was synthesized and then treated with alkaline phosphatase (ALP), which then cleaves the phosphate group to afford the gelator **24a** [60]. ALP is overexpressed in cancer tumors such as osteosarcoma and breast cancer cells, so they were examined in the study. Both compound **24a** and **24c** are able to bind to glucose transporter 1 (GLUT1) due to the presence of the sugar moiety and block glycolysis from happening within the cell [60]. In addition to that, after precursor **24c** is cleaved via ALP, the molecules can self-assemble on the cell surface forming a shielded network and disrupting cellular functions leading to cell death [60]. The researchers corroborated that responsive biomaterials can be utilized as an effective method for cancer treatment.

### 2.3. D-Mannose, D-Arabinose, D-Galactose, Derivatives as LMOGs

**Figure 19:**
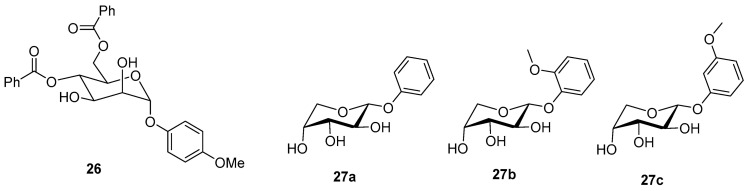
Structures of D-mannose derivative **26** and several arabinose derivatives **27** [61,62].

Pal et.al. reported a mannose-based low molecular weight organogelator (LMOGs) **26**, which exhibited significant self-healing capabilities as well as phase-selective aqueous-organic gelation [61]. It successfully gelled various organic solvents including several fuels, alcohols, aromatic solvents, halogenated solvents, and oils. Self-healing properties were observed in gels formed from almost all of the solvents tested. As shown in Figure 19, several arabinose glycosides were synthesized and analyzed by Pathak et al. [62]. Compound **27b** was able to form gels in benzene, petrol, diesel and crude oil, this gelator was then used for an oil spill clean-up experiment.

**Figure 20:**
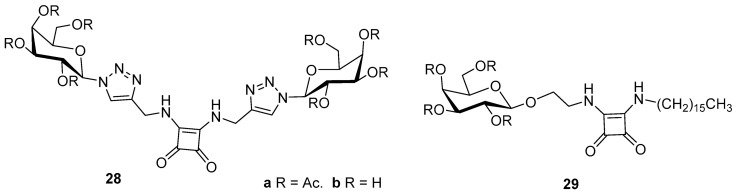
Structures of novel glycosyl squaramide supramolecular gelators [63].

Galactose derivatives have also been generated and studied for their gelation properties. Squaramides have been studied extensively in supramolecular chemistry as they contain both hydrogen bonding acceptors/donors and aromaticity. Ramos et al. combined galactosyl moieties with squaramide groups, triazole linkages and long aliphatic chains to create the first examples of glycosyl squaramide based LMWGs, as shown in Figure 20 [63]. The symmetrical triazole linked derivatives **28a** and **28b** were not very good gelators, only forming weak gels/aggregates in ethyl acetate and ethanol. On the other hand, the amphiphilic derivative **29a** performed very well in ethyl acetate, acetonitrile, ethanol and 1:1 aqueous ethanol mixture with fairly low minimum gelation concentrations [63]. After the removal of the acetyl groups under basic conditions, the amphiphilic derivative **29b** was found to be a supergelator in a 1:1 aqueous ethanol solution with a minimum gelation concentration of 0.1% w/v [63]. This study paved the way for the design and synthesis of a new class of supramolecular materials, which have a wide range of potential applications.

### 2.4. Glyconamide Derivatives by Modification at the Anomeric Position

Besides the cyclic sugar acetals, various linear derivatives have been prepared by functionalization at the C-1 anomeric position. Another major class of derivatives was prepared by the derivatization of glyconic acid or glyconolactone by the formation of various amides and esters. Several effective LMWGs have been obtained and these are discussed in the next section.

**Figure 21:**
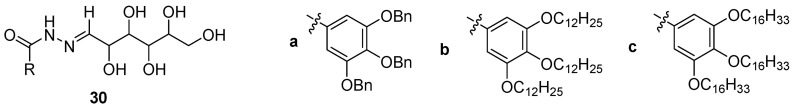
Structure of poly aryl ether glucose cored dendron-based LMWGs [64].

Kannan et al. designed and synthesized poly aryl ether and poly alkyl ether glucose cored dendron-based LMWGs (Figure 21) [64]. The results showed that there is a high tendency for the poly aryl ether dendrons to form strong gel system. These self-assembled systems were characterized using scanning electron microscopy (SEM), transmission electron microscopy (TEM), rheology, and powder X-ray diffraction (XRD). The dense fibrous network of the gels suggested that these gels would be ideal for the controlled release of various molecules.

**Figure 22:**
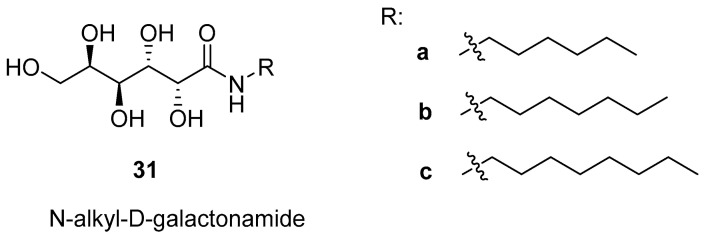
Galactonamide based gelators [65,66,67].

Several galactonamides **31a–c** were synthesized from galactonolactone with hexylamine, heptylamine, and octylamine (Figure 22). They gave similar gelation properties in water, with the heptyl and octyl derivatives forming spontaneously upon cooling whilst the hexyl derivatives showed slow gel formation [65]. The gels appeared to form ribbon like microstructures and the minimum gelation concentrations were 0.5 wt % for the octyl derivative, 0.45 wt % for the heptyl derivative and 1 wt % for the hexyl [65]. Consequently, the gels were utilized for growing neuronal cells in 3D. The DMSO solution with N-heptyl-D-galactonamide (**31b**) formed injectable gels in a water bath [66]. In a later study, they showed that a DMSO solution of **31b** could be injected into a water bath to print a gel on a glass slide covered with a hydrophobic polycarbonate membrane [67].

**Figure 23:**
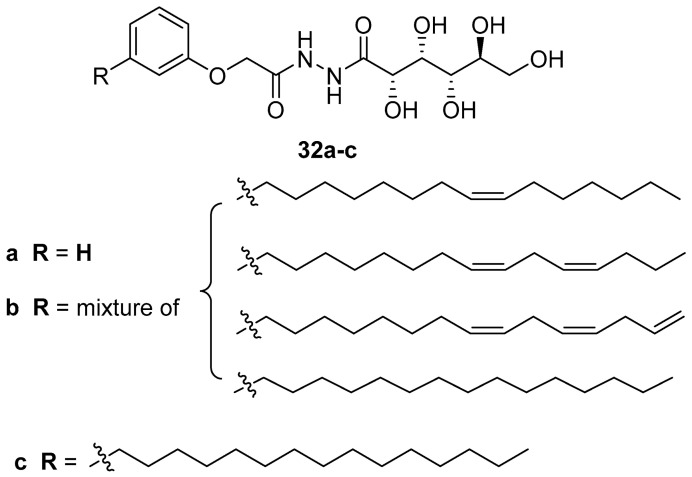
Gluconamide-based gelators [68].

The gluconamide-based amphiphiles in Figure 23 were generated from cashew nut shell liquid and δ-gluconolactone, which are rather easily available, renewable resources [68]. They synthesized three derivatives and only compounds **32a** and **32b** were able to form gels in the solvent systems that were tested [68]. Compound **32a** formed a gel in water at a concentration of 1.0 % *w*/*v* and **32b** formed gels in several organic solvents such as benzene, toluene, xylene and dichlorobenzene at a concentration of 0.8 % *w*/*v* [68]. Optical and field emission scanning electron microscopy (FESEM) images showed that they possess the typical fibrous network that is often observed for supramolecular gel. Additionally, the XRD data confirmed that the gelation mechanism was through hydrophobic interactions, hydrogen bonding and π-π interactions. The antibacterial properties of these glycolipids were tested and the results are discussed in the next section of the review [68].

**Figure 24:**
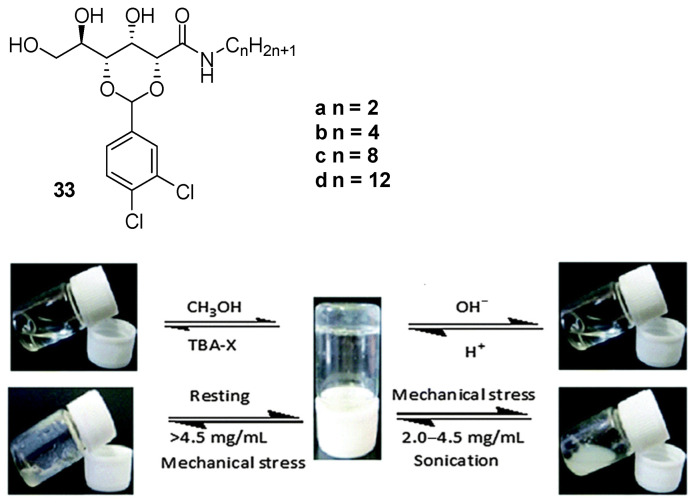
_D_-gluconic acetal-based gelators **33** and their response to different stimuli. Adapted with permission from [69]. Copyright 2016 The Royal Society of Chemistry.

Akin to the previous galactonamide-based gelators, other _D-_gluconic derived gelators containing 3,4-chlorobenzylidene acetal protecting groups and varying aliphatic chain lengths, displayed in Figure 24, were described by Song and his research group [69]. The acetal-based gelator with the eight carbon aliphatic chain **33c**, was the most versatile gelator, forming gels in a broad range of organic solvents and water mixtures. It could also form a gel in N-methy-2-pyrrolidone (NMP)/water (*v*/*v* 1:1) and chloroform at room temperature [69]. The chloroform gels made by this gelator underwent phase transitions in the presence of multiple stimuli. It was found to be sensitive to several anions, high pH, sonication and mechanical forces [69]. The gels were also versatile in their applications. The gel of compound **33c** in n-butyl acetate was able to extract pump oil from a biphasic mixture with saline water by spontaneously forming a gel at room temperature. The same gel also had remarkable mechanical strength as evidenced by its G′ value that was higher than 64 kPa with self-healing properties [69]. The chloroform gel of compound **33c** was sensitive to several anions. Solid tetrabutylammonium salts were placed on top of the gels and their response was observed overtime [69]. The gel containing I^−^ did not have a gel-sol transition, whilst those containing Br^−^_,_ AcO^−^, F^−^ and Cl^−^ changed to solutions after a while [69]. The authors believe the disruption in the gel phase may be due to the anions (X^−^) forming X⋯H--NCO hydrogen bonds, which interfere with the hydrogen bonds within the supramolecular network as evidenced by their ^1^H NMR spectroscopy results [69]. Adding a little extra methanol, based on the molar ratio to the gelator solutions, causes the reformation of the gels because it destroys the X⋯HNCO hydrogen bonds and allows for the hydrogen bonds in the gel state to be restored [69]. This gelator was so multi-faceted that it was even able to form a very robust transparent gel with moldable capabilities which they believe could allow for it to be employed in the manufacture of optical devices. This study presented a few incredible gelators with immeasurable potential for several applications.

**Figure 25:**
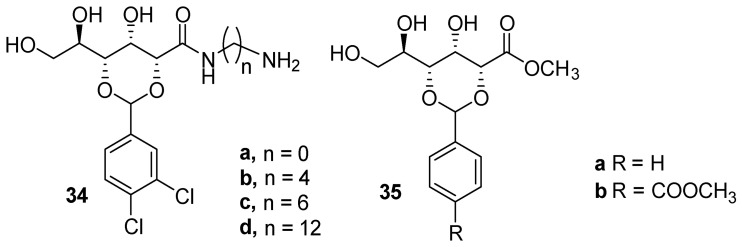
Structures of _D_-gluconic acetal-based gelators [71].

Liu et al. also studied two-component systems based on _D_-gluconic acetal derivatives with terminal amino groups in Figure 25. In this study, the terminal amino groups on the gelator molecules interact with various aliphatic acids to form two-component gels [70]. Based on the gelation tests results in organic solvents, there was an increase in the gelation properties as the aliphatic chain length increased in **34b–d** when compared to **34a**, which may be due to an increase in the van der Waal’s interactions [70]. The properties of **34c** surpassed the other gelators as it was labeled as a superior gelator in o-xylene and chlorobenzene, which had minimum gelation concentrations below 0.1 wt % [70]. The gelators were tested for the ability to form co-gels with several aliphatic acids shown in Figure 25. They found that **34a** was not able to form co-gels with any of the aliphatic acids, while **34b**–**d** were able to form co-gels in chlorobenzene, dioxane and o-xylene [70]. In addition to that, **34c** and **34d** also formed gels in octanol and toluene at very low concentrations [70]. The co-gels of **34c** with capric acid, myristic acid and stearic acid formed in chlorobenzene were very strong and showed self-healing properties. Additionally, the co-gel with stearic acid was highly transparent and could possibly be utilized in optical devices [70]. Compounds **35a**–**b** were also effective gelators in a wide range of organic solvents such as toluene, benzene, chlorobenzene, nitrobenzene and the three different types of xylene at extremely low concentrations ranging from 0.06–0.19 wt % [71]. The gelators from both articles were then tested for their phase-selective gelation properties which are discussed later in this article.

**Figure 26:**
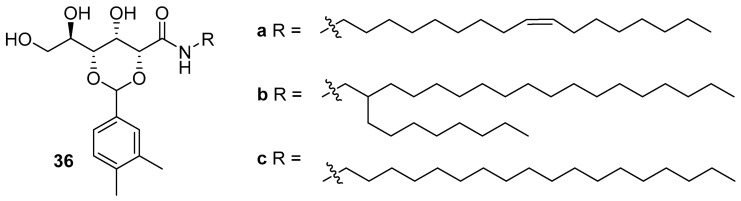
Chemical structures of other reported D-gluconic acetal based gelators [72].

The same group conducted research on other versions of _D_-gluconic acetal-based gelators shown in Figure 26. Compounds **36a–c** were effective gelators at low critical gelation concentrations of 0.05–0.36% *w*/*v* for a wide range of aliphatic and aromatic solvents, as well as petroleum products such as kerosene, gasoline and diesel [72]. When comparing gelation performance, compounds **36a** and **36b** generally had lower gelation concentrations which could confer that the existence of the irregular alkyl chains allowed for increased solubility in hydrocarbon solvents [72]. The authors concluded from their XRD, SEM and Fourier-transform infrared spectroscopy (FT-IR) studies that the abnormality in the alkyl chains may have influenced the formation of powders that allowed for the favorable gelation of oil due to increased interactions between the chains at the surface and the oil molecules [72].

### 2.5. Alditol Derivatives from Reduced Sugar Alcohols

Lai et al. found that 1,3:2,4-dibenzylidene-D-sorbitol (DBS) can form supramolecular gels in low-molecular-weight poly(ethyleneglycol) (PEG). The self-assembly of DBS in PEG was found to be significantly influenced by the addition of inorganic silica. In this study, they found that the addition of silica did not reduce the aromatic interactions between the phenyl rings of DBS; however, it did affect intermolecular hydrogen bonding between the DBS and PEG [73]. The reduction in the interactions between DBS and the solvent increased intermolecular interactions between DBS molecules, thus increasing self-assembly. Due to this, gel formation time and gel dissolution temperature increased with increasing amounts of silica. Polarized optical microscopy showed that the microstructures of these organogels have a spherulite-like morphology and small-angle X-ray scattering showed the spherulite-like morphologies contained lamellar packing [73]. Increasing the amount of silica in the gel increased the temperature of self-assembly and decreased the amount of nucleation sights, which led to larger spherulite sizes. SEM indicated that the addition of silica increased the diameter of DBS nanofibrils. This study showed how the self-assembling behavior of a gelator in specific solvents can be modified through the addition of inorganic silica, creating interesting hybrid materials.

**Figure 27:**
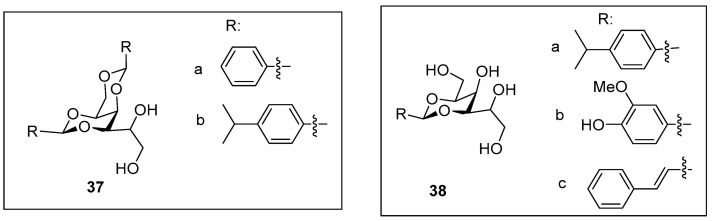
Structures of sorbitol diacetals **37** and monoacetals **38** [74].

Dizon et.al. designed and synthesized mono- and di-benzylidene protected sorbitol-based LMWGs [74]. These derivatives include 1,3:2,4-di(4-isopropylbenzylidene)-D-sorbitol (**37b**) and 2,4(4-isopropylbenzylidene)-D-sorbitol (**38a**), which are shown in Figure 27. Compound **37b** was able to form gels in most of the organic solvents and all of the ethanol-water mixtures; however, it was not a hydrogelator. On the other hand, **38a** did not form many organogels; however, it was found to be a good hydrogelator and formed gels in various aqueous salt solutions. The other analogs **38b** and **38c**, formed by replacing the isopropyl benzene with other substituents, were not gelators. Mixtures of **38a** and **37b** were found to have a lower minimum gelation concentration in ethanol-water solutions compared to the minimum gelation concentrations of the individual gelators. Rheological experiments also indicated the mixtures of **37b** and **38a** formed stronger gels compared to the gels from **37b**. These multicomponent gels could find applications as tunable soft materials in a variety of applications.

**Figure 28:**
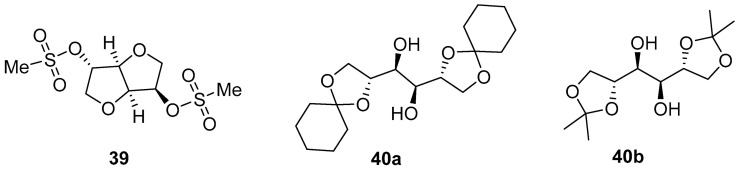
Structures of isosorbide derivative **39** and D-mannitol derivatives **40a–b** [75].

McNeice et al. found three sugar-based LMWGs capable of forming gels in ionic liquids and the structures are depicted in Figure 28 [75]. A total of 21 different ionic liquid gels were achieved using these gelators. These include imidazolium, pyridinium, phosphonium and ammonium-based ionic liquid gels. They explored the role of hydrogen bonding and steric effects in self-assembly when varying the cationic and anionic components of the ionic liquids. The larger the size of the cationic component, the larger the amount of gelator required for gels to form. Bulky ionic liquids were found to disrupt gelation in comparison to ionic liquids that contain a single alkyl chain. This study also found that ionic liquids with strong hydrogen bond donating and accepting components did not prevent gelation, indicating a strong interaction between each other rather than interfering with gelator self-assembly. While all three gelators successfully formed gels in ionic liquids, gelator **40a** formed gels in almost all of the ionic liquids tested. Expectedly, the ionic liquid gels showed higher thermal stability compared to the ionic liquids themselves. Reversible ionic liquid gels such as these have potential uses in various electrochemical applications.

**Figure 29:**
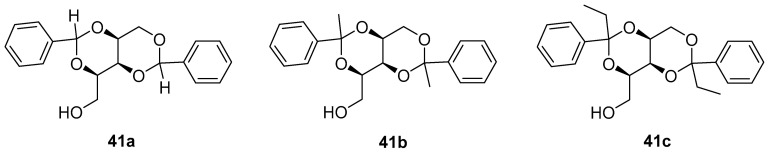
Xylitol based organogelators [76].

Raju et al. reported three xylitol based organogelators **41a**–**c** as shown in Figure 29 [76]. They were found to form gels in a variety of organic solvents and crude oils. Compound **41a** was the best gelator in the series for crude oil gelation, forming gels with the highest mechanical strength. Using the gelator in powdered form did not lead to the formation of gels in biphasic solutions of crude oil and water; however, adding a solution of the gelator in toluene did. Gelation for various refinery fractions and crude oil occurred within two minutes. After removal of the solidified oil layer from the biphasic solutions, vacuum distillation was carried out to recover the oils and the gelators. The recovered gelators were reused and underwent five cycles with minimal loss in efficiency.

### 2.6. Glycosyl Triazole Derivatives as Molecular Gelators

Heterocycles, including triazoles, have been utilized as functional groups in low molecular weight gelators. The triazole moiety provides many advantages for the gelation properties; it can be easily accessible through click reactions, it forms π-π interactions and its structure can accommodate hydrogen bonding through its hydrogen bonding acceptors and donors [77,78].

#### 2.6.1. C-1 Triazole Glycoside Derivatives

**Figure 30:**
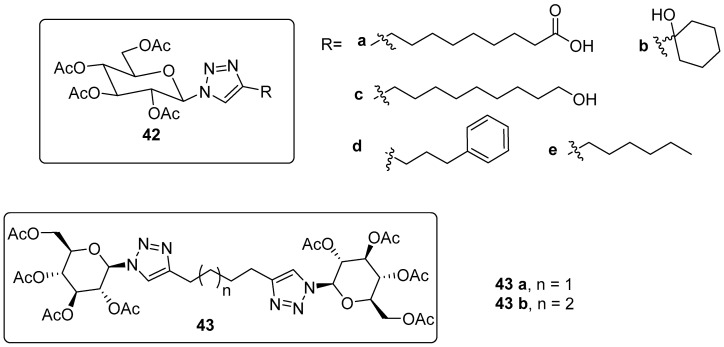
Structures of glucosyl triazole-based gelators [39].

**Figure 31:**
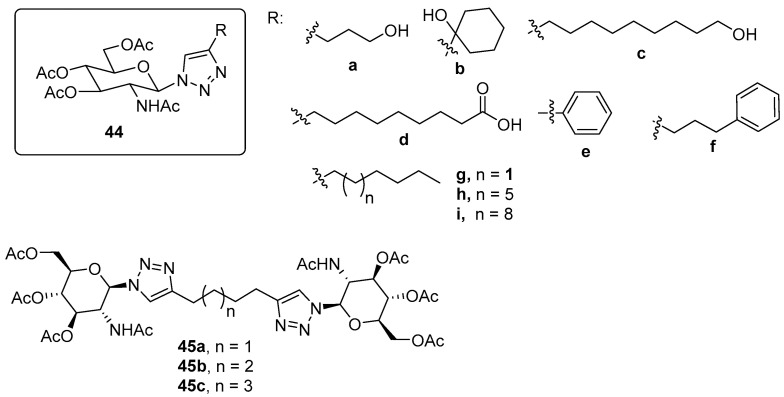
D-glucosamine triazole derivatives synthesized by the copper (I) catalyzed azide alkyne cycloaddition reaction [36], n represents the number of CH_2_ unit.

**Figure 32:**
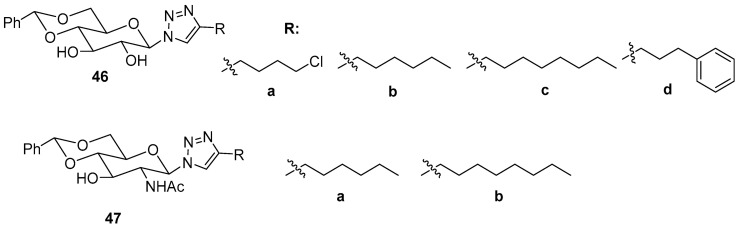
Structures of the benzylidene acetal protected β-triazolyl glucosides as gelators [79].

The Wang group has designed, synthesized and studied the self-assembling properties of a series of sugar triazoles using D-glucosamine, D-glucose, and D-maltose triazole derivatives. The 1,2,3-triazoles were synthesized typically using a sugar azide with an alkyne via the Cu (I) catalyzed azide/alkyne cycloaddition reaction (CuAAC). The compounds have been screened for their gelation properties and several efficient low molecular weight gelators were obtained, with many of them forming hydrogels [36,39,40,41]. The general structures of these compounds are shown below in Figure 30, Figure 31 and Figure 32.

The earlier research involved the incorporation of the triazole moiety at the anomeric position using peracetylated glucosyl headgroup Figure 30 [39]. Several analogs were gelators in aqueous mixtures and polar solvents, and a hydrogel was formed by the long chain alcohol derivative **42c** [39]. The carboxylate **42a** formed a gel in mixtures of DMSO and water at 1:1 and 1:2 ratios. The hydrophobic alcohol derivatives **42b** and **42c** were effective gelators too. The derivatives without polar substituents **42d** and **42e** were only able to form gels in EtOH:H_2_O (*v*/*v* 1:2) and in isopropanol. The dimers **43a–b** were more effective gelators, with **43a** forming gels in toluene and ethanol as well as aqueous mixtures of ethanol and DMSO and **43b** forming gels in six different tested solvents [39].

Using the D-glucosamine starting material, a library of 16 peracetylated D-glucosamine triazole derivatives were synthesized and analyzed. Effective LMWGs, including seven hydrogelators, were obtained [36]. A few representative structures are shown in Figure 31. The dimers **45b and 45c**, with six and seven carbon alkyl spacers between the two triazole moieties, also formed hydrogels [36]. Compounds **44a–d** and **44f–h** were hydrogelators that also gelled in aqueous DMSO and ethanol mixtures [36]. Derivatives **44a–b** were gelators in toluene below 2.0 mg/mL. Overall, the glucosamine triazole gelators (Figure 31) were better hydrogelators than the glucose-based triazole and typically had lower CGCs for the monomers [36,39].

To understand how the functional groups influence the molecular assemblies and also to find more efficient LMWGs, Chen et al. synthesized and studied 4,6-O-benzylidene acetal protected sugar triazoles **46** and **47** (Figure 32) [79]. These 4,6-O-benzylidene acetal protected glycosyl β-triazoles were synthesized from the corresponding azides through click chemistry. Several gelators were obtained from the synthesized triazole derivatives. The aliphatic derivatives **46b** and **46c** were mainly gelators for pump and engine oil; a few also formed gels in aqueous mixtures of DMSO and ethanol. The aromatic derivative **46d** formed a gel in DMSO:H_2_O (*v*/*v* 1:2) at 6.7 mg/mL. The triazole derivatives synthesized from N-acetyl D-glucosamine, **47a** and **47b** did not form gels in pump oil, instead, they formed gels in toluene, and aqueous mixtures of ethanol and DMSO. The presence of the 4,6 benzylidene acetal functional group seems to diminish the gelation tendencies for some triazolyl glycosides.

**Figure 33:**
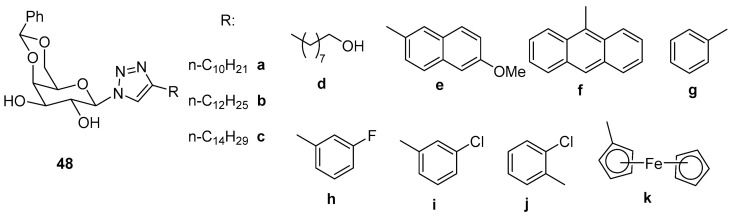
Structures of 4,6-O-benzylidene–galactopyranoside gelators [80].

Krishnan et al. reported the synthesis and gelation properties of a series of 11 4,6-benzylidene acetal protected β-triazole derivatives from D-galactose (Figure 33) [80]. These compounds were tested in hydrocarbon solvents and oils with CGCs in the range of 0.10 to 3.58 wt %. The long-chain triazole derivatives **48a–c** were gelators for only oils and tetra-ethyl-orthosilicate. The aromatic derivatives formed gels in organic solvents, but not in oil. An anthracene-containing gelator **48f** showed interesting fluorescent properties and could have applications in various fields [80].

**Figure 34:**
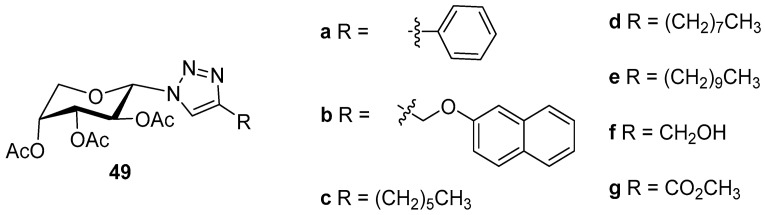
Structures of a series of triazolylarabinoside derivatives [81].

A series of triazolylarabinoside derivatives were also reported and two of these derivatives shown in Figure 34 were effective organogelators [81]. Derivatives **49a** and **49g** were found to be supergelators in non-polar solvents such as petrol, diesel and kerosene, with CGCs below 1% *w*/*v* making them ideal candidates for applications in solidifying crude oil and crude oil distillates. The solidified oil layers were scooped out and distillation was used to recover the oils [81]. After distillation, the gelator was purified via column chromatography (SiO_2_) with an 80–90% recovery.

#### 2.6.2. Carbohydrate Derivatives Containing Triazoles (not Anomeric Positions)

**Figure 35:**
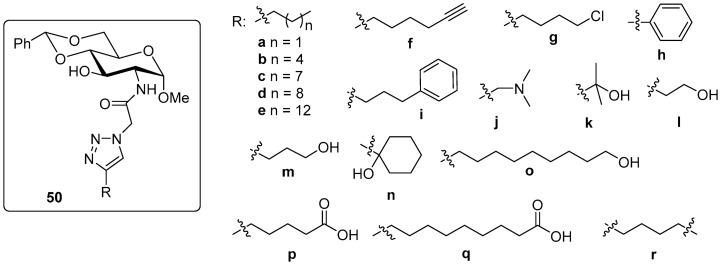
Other gelators with a triazole backbone [40], n stands for the number of repeating CH_2_.

From the peracetylated glucosamine triazole derivatives, several effective LMWGs were obtained. Besides the anomeric positions, the triazole unit can also be introduced to other positions of the sugar gelator template. Wang et al. reported the introduction of the triazole group at the 2-acyl position on glucosamine as seen in the compounds in Figure 35 [40]. The structural modification resulted in stronger gels, possibly due to additional hydrogen bonding from the amide and π-π stacking by the triazole component [40]. The hypothesis proved to be true with the series producing eleven effective hydrogelators at low concentrations of 0.15–1.0 wt %. Most of the analogs were also good gelators for 33% aqueous mixtures containing ethanol or DMSO [40]. Derivatives **50a–r** were good gelators in ethanol, isopropanol, aqueous mixtures of ethanol or DMSO at ratios of 1:1 and 1:2. The propyl compound **50a** was the best gelator in water with a concentration of 1.5 mg/mL [40]. Several other hydrogels were obtained from **50f–g**, **50i–m** and **50o–q** at relatively low concentrations [40]. Overall, the compound that gave the lowest gelation concentration was compound **50b** in DMSO: H_2_O (*v*/*v* 1:2) at 1.0 mg/mL [40]. The results of this study confirms that the D-glucosamide triazole structure is effective for designing soft materials based on sugars [40].

**Figure 36:**
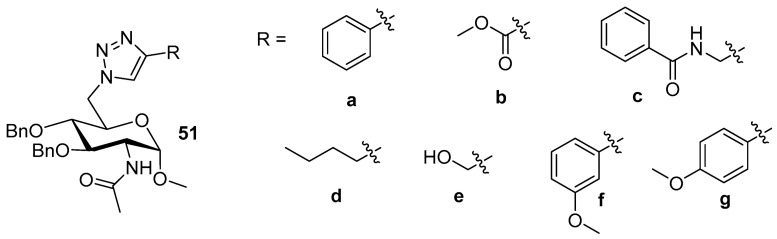
Triazole linked N-acetylglucosamine-based gelators [82].

The triazole functional group was also introduced to the C-6 position of N-acetylglucosamine and several derivatives were found to be effective gelators for various organic solvents and oils [82]. As shown in Figure 36, compounds **51a–b** and **51f** were capable of phase selective gelation in three different types of crude oils. While the gelators were not able to gel crude oil in the powdered form, having ethyl acetate as a carrier solvent did not lead to gelation. Using this method, phase selective gelation occurred at room temperature within 15 min of the addition. In additional studies, mesitylene gels formed by compound **51a** were found to remove rhodamine B and crystal violet from aqueous solutions. The usage of non-toxic carrier solvents such as ethyl acetate to deliver and disperse gelators in crude oil spills may be useful in larger scale clean up applications.

#### 2.6.3. Glucosyl Triazole and Nucleoside Hybrid System

**Figure 37:**
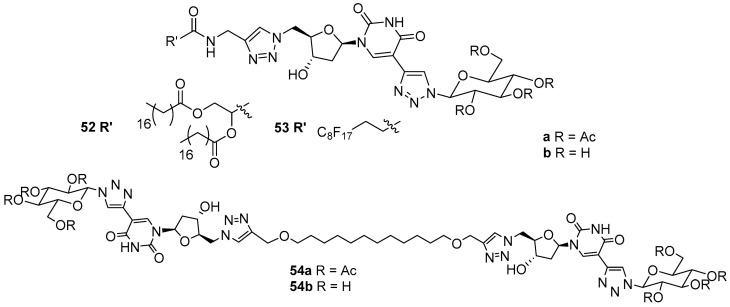
Uracile based glycosyl-nucleoside-lipids amphiphiles and bolaamphiphiles [83].

The glycosylated uracil-based gelators in Figure 37 were synthesized and characterized [83]. Of the six reported compounds, **53b** and **54b** were not soluble in any of the tested solvents and **54a** was soluble in some but not able to form a gel. Organogels were seen from the other derivatives in chloroform, ethanol, methanol, acetonitrile and toluene. Optical imaging showed the microstructures of gels formed by **52a** in methanol and ethanol at 2% were composed of long intertwined fibers. The rheological studies confirmed a viscoelastic gel was formed; the gel expressed thixotropic behavior as it could rebound back to the gel state after being exposed to high strain [83]. These uracil derivatives add to the existing knowledge on nucleoside-based gelators in the literature and show good promise for being further investigated as injectable biomaterials.

**Figure 38:**
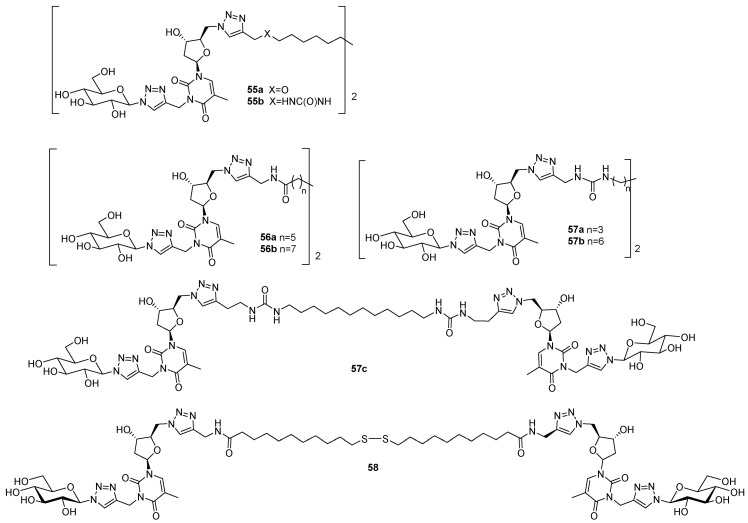
Structures of amide and urea containing bolaamphiphiles [84,85].

Bolaamphiphiles with sugar moieties generated from nucleosides have also been studied in the literature and a few representative molecules are shown in Figure 38. The bolaamphiphiles **56a** and **57b** in the article were soluble in water, whilst **56b**, **57a** and **57c** were able to form hydrogels at a minimum gelation concentration of 0.14, 0.10 and 0.21% *w*/*v* respectively [84]. The gels had the typical dense fibrous network and hydrogen bonding was proven to be the major non-covalent force driving the self-assembly mechanism [84]. Of all the hydrogels, the one formed by compound **57a** was the best candidate for injectable applications because of its mechanical properties. The gels of **56b**, **57a** and **57c** were loaded with a cyanin dye and implanted in mice to investigate dye release and gel degradation. The gel formed by **57a** was still intact up to three weeks after the gels were injected under the skin of the mouse [84]. The gel was also non-immunogenic and non-toxic which makes it an ideal biomaterial for in vivo applications [84].

Bansode et al. designed and synthesized glycosylated nucleoside-based bolaamphiphilic LMWGs [85]. A disulfide functional group, linking the amphiphiles allows for in vitro selective and sustained release of thiol-containing proteins. Stable hydrogels were formed from the glycosylated nucleoside-based bolaamphiphile (GNBA) **58**. The resulting hydrogels were shown to have thixotropic properties, characterized via rheological studies, making them candidates for extrusion-based gel injections [85]. The rationale behind including the disulfide functional group in the middle of the aliphatic chain is to introduce a functional group capable of thiol-disulfide exchange reactions. This allowed hydrogels formed with these disulfide GNBA gelators to be functionalized with thiol-containing proteins, which could then be slowly released in vitro [85]. This study reveals the first example of a supramolecular gel formed by a LMWG that exhibits sustained release through a disulfide reshuffling mechanism.

### 2.7. Disaccharide Derivatives and Glycoclusters

#### 2.7.1. Disaccharide Derivatives

**Figure 39:**
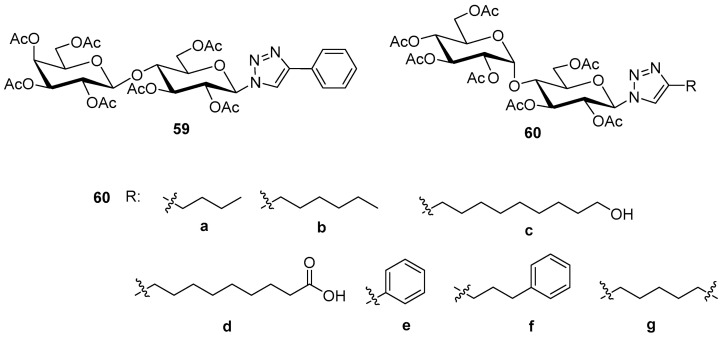
Structures of peracetylated lactosyl and maltosyl triazole derivatives [41].

To further explore the sugar structure and gelation correlations, a series of disaccharide triazole derivatives were synthesized and their effectiveness as LMWGs were studied [41]. Nine peracetyl lactosyl triazole derivatives and thirteen peracetyl maltosyl triazole derivatives were synthesized [41]. Figure 39 shows the structures of the compounds that formed gels. The lactosyl derivatives typically did not form gels, except compound **59**, which formed gels in EtOH:water (*v*/*v* 1:1) and DMSO:water (*v*/*v* 1:1). This may be attributed to the phenyl group’s additional π-π interactions allowing for the molecule to self-assemble into fibers in polar solvents [41]. On the other hand, the maltose-based disaccharides were effective gelators that formed gels in aqueous mixtures of DMSO or ethanol as well as isopropanol and ethanol. Compound **60d** was an efficient gelator in aqueous mixtures of DMSO or ethanol with concentrations ranging from 2.0–5.0 mg/mL. The phenyl derivative **60e** formed a stable gel in ethanol as well as the aqueous mixtures with concentrations ranging from 2.0–6.7 mg/mL [41]. The disparities with the gelation properties of the two classes of disaccharides could possibly be associated with the structural pattern of the sugar groups [41].

**Figure 40:**
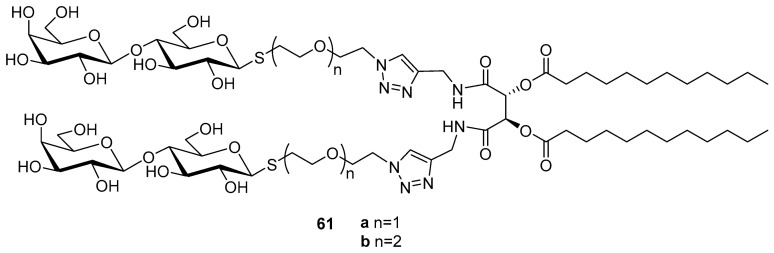
Structures of thiolactose-based amphiphilic gelators [86], n is the number of repeating unit.

Cano et al. designed and synthesized two thiolactose-based amphiphilic LMWGs [86]. The two amphiphiles were built from a di-lauroyl-L-tartaric acid scaffold and only differed in the length of the ethylene glycol linker (Figure 40). The lactose-containing amphiphiles were capable of forming colloidal systems in water. The amphiphile with the shorter linker **61a** formed a hydrogel at a concentration of 0.1% *w*/*v*. Circular dichroism of the self-assembled aggregates showed a chiral inversion for amphiphile **61b** compared to amphiphile **61a**, indicating the length of the ethylene glycol linker significantly affects the chiral self-assembly of these molecules. The ability of the amphiphiles to bind to peanut agglutinin (Pna) lectin was also studied using a turbidimetric method and agglutination was observed in both cases. The structural parameters impacting amphiphilic self-assembly were explored in this study, contributing to knowledge that could be used in rationally designing nanostructures which expose specific sugar ligands.

#### 2.7.2. Branched Glycoclusters from Glycosyl Azides.

Dendritic compounds are unique types of molecules which can possess useful features not present in non-dendritic compounds. The creation of glycoconjugates with well-defined structures and sizes in between small molecules and polymers is a pathway for discovering new materials with desirable physical and mechanical properties. Branched systems or dendritic compounds can be considered as lower generation glycoclusters with possible multivalent effects. Incorporating catalysts into these glycoconjugates will be useful in the synthetic field. The design rationale of these compounds is based on the concept of multivalency, where individual functional units may perform synergistically when tethered. Dendritic structures have been shown to be effective in the generation of LMOGs [87,88]. Dendrimers have the advantage of uniform molecular weight compared to polymers and can also allow one to introduce desired functionalities into the structure. Typically, the formation of gels using LMWGs requires heating and/or sonication, as the gelation is generally not spontaneous. Another potential issue for LMWGs is that the gels may not be as stable as polymer gels. This could be advantageous for certain applications, while posing a problem in other situations. Dendritic molecules utilizing multiple low molecular weight gelator subunits as their monomeric building blocks can lead to new materials that have enhanced stability, improved functionality, and better flexibility, compared to low molecular weight gelators.

**Figure 41:**
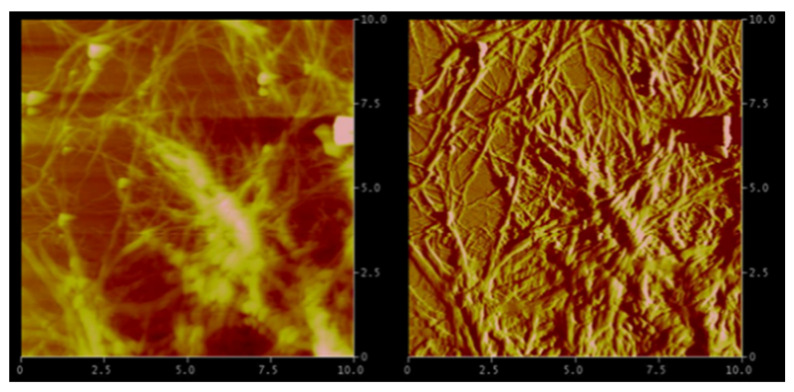
AFM images of a 10 mg/mL hydrogel from **62** (left, height image; right, phase image) [87].

**Figure 42:**
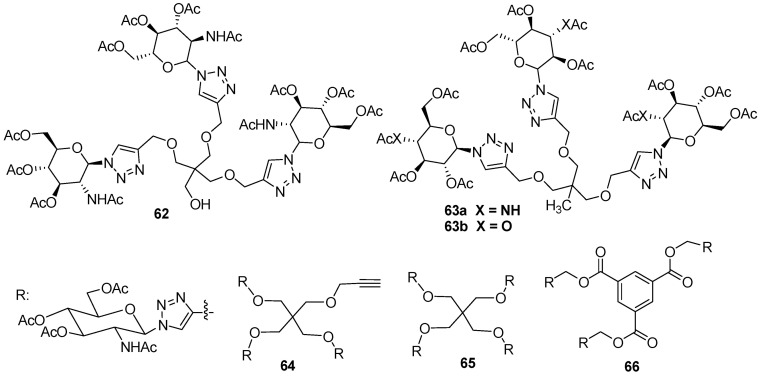
Structures of several glycoclusters that are effective LMWGs [87,88].

We have designed and synthesized a series of mono-, di-, tri- and tetra-functionalized pentaerythritol cored glycoconjugates to explore the effect on supramolecular self-assembly when the number of branches in the glycocluster is increased [87]. In this study, a pentaerythritol core was linked to peracetylated sugar units through copper catalyzed azide alkyne cycloadditions (CuAACs). Increasing the number of triazole-linked branches containing peripheral sugar units was found to enhance gelation properties. Several of the trivalent and tetravalent glycoconjugates were found to be effective organogelators and hydrogelators as shown in Figure 41 and Figure 42. The trimeric glycoconjugate **62** was found to be an effective hydrogelator, which was capable of the encapsulation and time-dependent sustained release of naproxen sodium, riboflavin, and vitamin B_12_. The results of this study showed that linking three to four small molecules to a central core to create trimeric and tetrameric conjugates can be a rational approach to designing supramolecular gelators.

**Figure 43:**
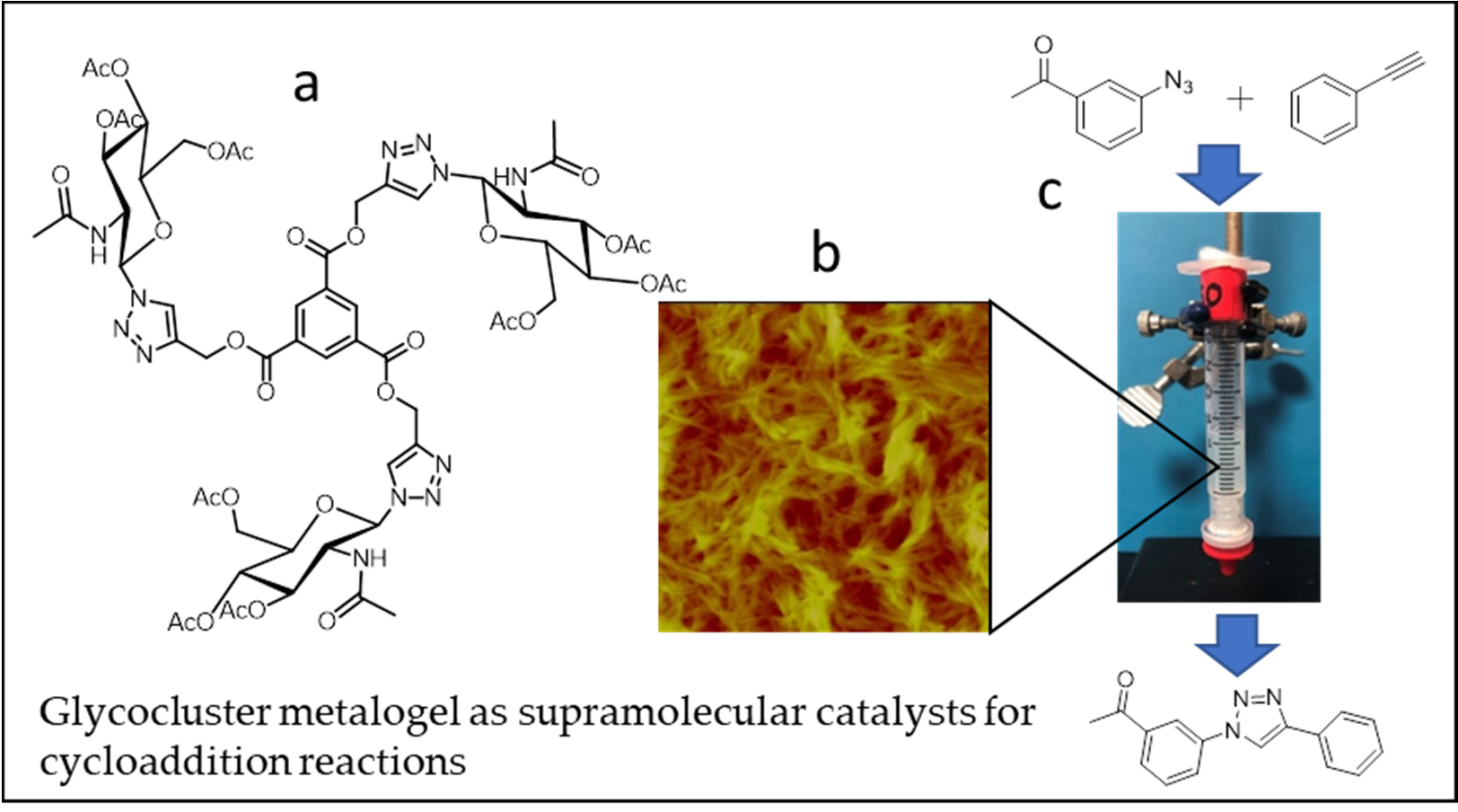
(**a**) Structure of the trimer glycocluster **66**; (**b**) AFM image of the gel formed by **66** in 1:2 ethanol/water (*v*/*v*); (**c**) A copper metallogel column formed by **66** in 1:2 ethanol/water (*v*/*v*) was transferred to a syringe, which was used as a reaction vessel. This figure is adapted from [88] with modifications.

More recently, we have designed and synthesized twelve dendritic glycoclusters containing three, four and six glycosyl triazole branches [88]. The clusters with fewer branches were found to be effective supramolecular gelators and the hexameric glycoclusters were found to efficiently accelerate copper sulfate mediated cycloaddition reactions. The glycoconjugates **63** [88] and **64–66** were also effective gelators for alcohols and aqueous mixtures (Figure 42). Combining the efficient trimeric gelators and hexameric catalysts led to the formation of stable co-metallogels that could be used as supramolecular catalysts. Biphasic metallogel reaction mixtures and metallogel columns were utilized to carry out several CuAAC and isoxazole synthesis reactions, using a variety of substrates as shown in Figure 43. The metallogels were successfully reused for up to six reaction cycles, showing the reusability of these soft catalytic materials [88]. The gels formed in this study may have applications in other catalytic reactions using copper ions, as well as catalytic reactions that require alternative metal ions.

### 2.8. Nucleoside/Nucleotide-Based Gelators

Nucleosides/Nucleotides are also important molecules that have been studied in supramolecular chemistry for their self-assembling properties. Their molecular structure includes a ribose sugar with purines or pyrimidines that can form intermolecular attractions via many mechanisms including π-π stacking, electrostatic interactions and hydrogen bonding [89]. Many of the gelators that have been reported in this category are generally prepared by modification of the ribose sugar or the nucleobase moiety to introduce lipophilic linkers that provide some amount of hydrophobic character for gel formation [89]. The gelators have been found to form gels in a wide variety of solvents ranging from water all the way to non-polar solvents like hexanes [89]. There have been reports of the gelators containing all four nucleobases, but guanidine remains one of the most common and attractive molecules for these gelator systems. This is probably because guanidine has the potential to form extended networks of 2D ribbons and G-4 wires that are more biochemically significant [89]. In this section of the review, a few of the more recent systems in this group will be highlighted. Nucleic acid based supramolecular materials, have been widely studied for biomedical and technological applications. Recently researchers have emphasized the molecular design, synthesis, supramolecular properties, physicochemical behaviors, and applications on these supramolecular materials. The design and the study of nucleolipids are the main focus of this section. The supramolecular materials generated by nucleic acid-based lipids open new opportunities for biomedical applications [90]. Nucleic acids utilized as self-assembling organogelators will be discussed in detail in the following examples.

#### 2.8.1. 2-Deoxy Ribose Derivative as Gelators

**Figure 44:**
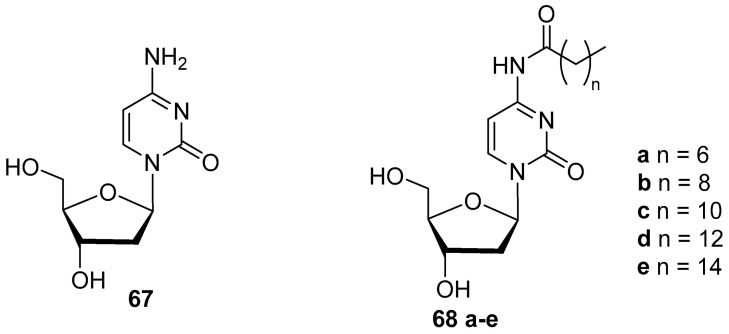
Cytosine and cytosine-based gelators [91,92].

Angelerou et al. reported a cytosine-based supramolecular gelator, the *N*^4^-octanoyl-2′-deoxycytidine **68a**, as shown in Figure 44 [91]. The *N*^4^-octanoyl-2′-deoxycytidine was able to form gels in water and mixtures of water with organic solvents such as methanol, but not in pure organic solvents [91]. The gel formed by compound **68a** in ethanol/water 20:80 *v*/*v* % was transparent in appearance and had increased fluorescent properties when compared to the solution in methanol and its parent molecule **67**. The researchers believe that the enhanced fluorescence is due to the self-assembly via π-π stacking which led to aggregation-induced emissions that basically blocked the gelator’s chromophore from solvent-induced quenching to strengthen the fluorescence signal [91]. They discussed several detailed studies on the self-assembling properties of the gelator and concluded that rationally designed nucleoside-based gelators have great potential to be utilized in drug delivery systems [91]. They have further studied a series of 2′-deoxycytidine-based gelators with different acyl chain lengths, compounds **68b–e** as shown in Figure 44 [92]. These four compounds all formed gel films on piranha cleaned silicon wafers (OH surfaces). They analyzed the structures of the gel films using time-resolved grazing incidence wide angle X-ray scattering (GIWAXS) on gel films [92]. The study showed that the size of the hydrophobic alkyl chains directly affects the diameter of gel fiber, but the surface materials on which the gel was deposited affect the aggregation or bundling of the fibers.

**Figure 45:**
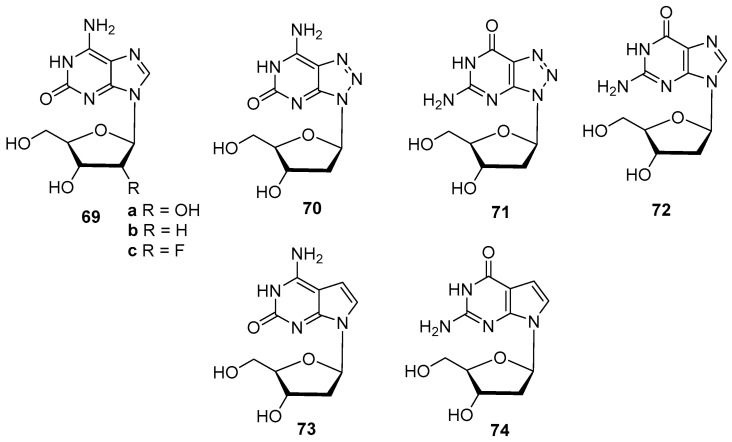
Nucleoside derivatives based on guanosine analogs [93].

Other guanosine-based gels were reported in the literature including the compounds portrayed in Figure 45. The vial-inversion method was used to validate the gelation properties of the compounds in the presence of different salts of alkali metal ions [93]. Compound **71** was not able to form a gel in any of the tested metal ions whilst compounds **69b**, **70** and **72** successfully formed gels with several of the tested metal salts [93]. The 8-Aza-2′-deoxyisoguanosine derivative **69b** illustrated strong fluorescence under a UV lamp at a wavelength of 366 nm and the sol to gel transition was also observed. The gel was able to re-form when the irradiation was halted [93]. In addition to that, the latter gel was also responsive to pH changes and temperature, which makes for a useful candidate in many fields such as nanomedicine and chemistry [93].

**Figure 46:**
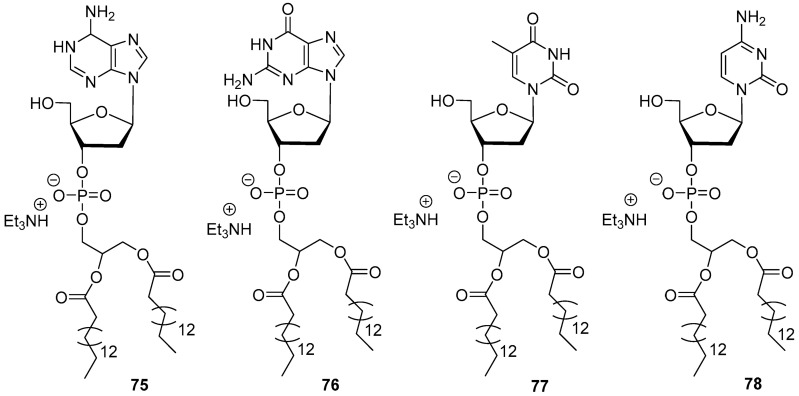
Cytidine- and guanosine-based nucleotide–lipids [94].

A phosphoramidite approach was employed to synthesize four nucleotide-lipids containing both purine and pyrimidine bases as shown in Figure 46 [94]. These nucleotide-lipids were able to form hydrogels. The gels responded differently to the addition of sodium chloride (NaCl) and the major compounds highlighted in the paper were the guanosine derivative **76** and the cytosine analog **78**. The cytosine lipid was able to form a stable hydrogel in the presence of NaCl, whilst the guanosine lipid did not need NaCl to stabilize the hydrogel [94]. Dynamic light scattering (DLS), circular dichroism (CD) and differential scanning calorimetry (DSC) studies showed that the supramolecular structure of the respective gelators was dependent on the specific nucleobase within the gelator and other conditions. The gels of compounds **76** and **78** were further exploited as drug delivery systems for diazepam which was slowly released from the gel matrix over time [94]. However, in the presence of NaCl the drug was released at a faster rate from the guanosine gel than the cytosine derivative indicating that drug release can be regulated based on the type of the nucleotide and in the presence of salts [94]. The gels show good potential to be utilized in controlled delivery systems for biomedical applications.

**Figure 47:**
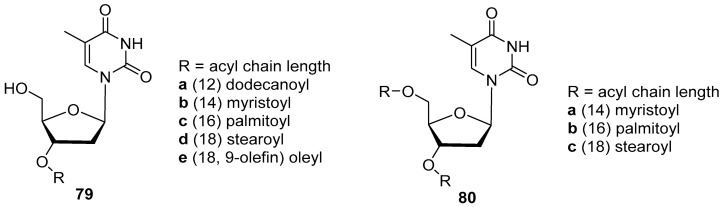
Structures of thymidine-based gelators [95].

A series of thymidine-based nucleolipid derivatives from 2-deoxyribose were synthesized and their gelation properties studied (Figure 47) [95]. These nucleolipids include the 3′-O-monoesters **79** and 3′,5′-O-diesters **80**. The long fatty acid tail allows for the self-assembling process and the NH groups and oxygen atoms allow for hydrogen and metal ion bonding [95]. The mono substituted derivatives **79a–e** were not effective gelators in polar organic solvents, but several were able to form gels in mixtures of water with DMSO, methanol, toluene and DMF [95]. The oleyl derivative **79e** was the only compound that did not form any gels. Contrary to that, the di-substituted gelators **80a–c** did not rely on the addition of water to form gels in the tested solvents. The gelators **80a–c** gelled DMSO and derivatives **80b** and **80c** were able to form gels in acetonitrile as well [95]. This indicates that the length and number of fatty acid chains can be utilized to tune the gelation properties of the molecules. Surface property studies of thin films developed from the toluene-water and methanol-water gels of compound **79c** confirmed that the gel surface could switch from being extremely hydrophobic to hydrophilic giving rise to functional gels [95]. Another important finding was the response of the gels to metal ions such as mercury. The mercury, lead, silver, zinc and cadmium metal ions were added to the DMSO solution of compound **80c**, the solution with all the metal ions except mercury were able to form a gel indicating that the gel may be used for ion sensing applications [95].

#### 2.8.2. From Ribose Derivatives

**Scheme 1:**
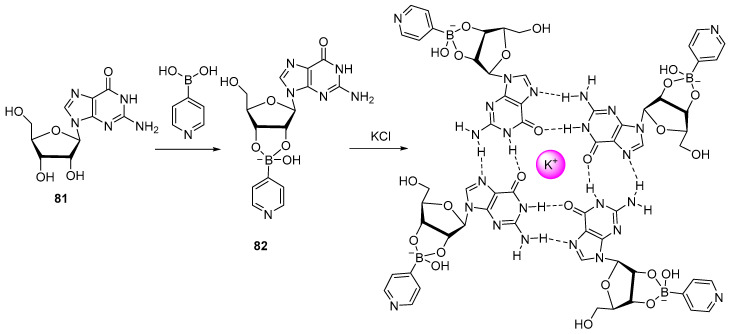
The synthesis and self-assembly of a nucleoside-based gelator with potassium ion [96].

As previously described, nucleosides can be utilized for supramolecular gelators; however, self-assembly of the molecules has underlying rules that are poorly understood. Even though guanosine based gelators are known to form highly ordered assemblies, they fall short when it comes to stability [96]. To address this issue, Li et al. have discovered a hydrogel shown in Scheme 1 below, that was formed by mixing guanosine (4% *w*/*v*) with pyridine-4-boronic acid (PyB) in aqueous potassium chloride (0.14 M) at a molar ratio 1:1:1 [96]. The hydrogel was transparent with a dense fibrous network and the potassium chloride particles were randomly dispersed in it. The guanosine-based hydrogel was stable for quite a few months and the gel-sol transition could be manipulated by changing the concentrations of the different components. In addition, the hydrogel exhibited polymer-like swelling behavior in water and high ionic conductivity. The electrical impedance spectroscopy studies confirmed that the gel could be utilized in electrochemical biosensing platforms; the xerogel also has the capacity for phase-selective gelation for toluene and water [96].

**Figure 48:**
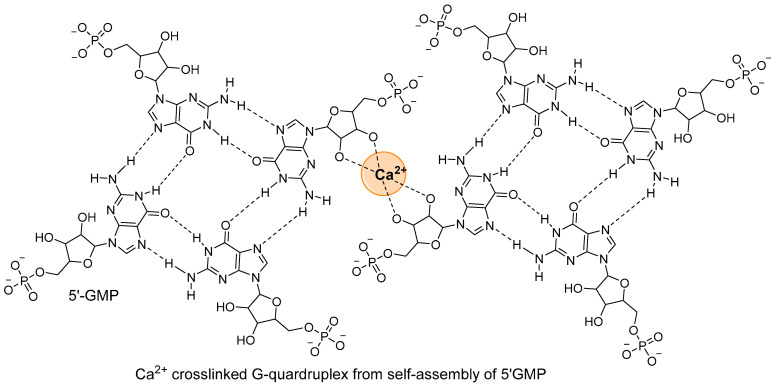
Guanosine monophosphate and the self-assembled G-quadruplex with Ca^2+^ [97].

**Figure 49:**
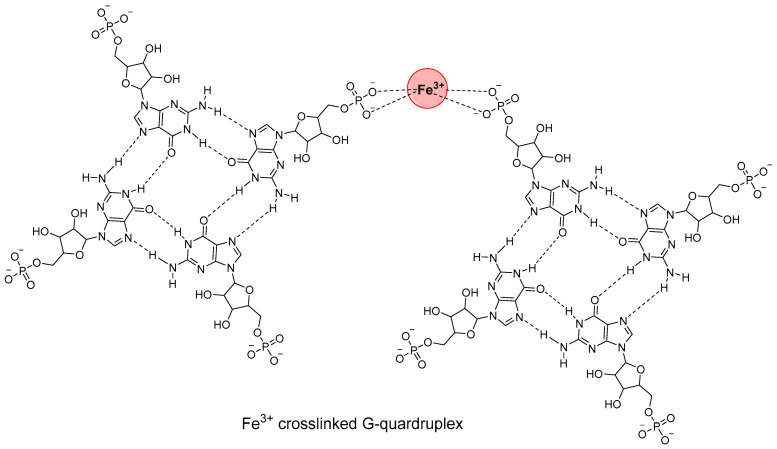
The self-assembled G-quadruplex with Fe^3+^ [97].

Thakur et al. developed an Fe^3+^ and Ca^2+^ dual cross linked guanosine monophosphate hydrogel capable of pH-responsive zero-order release of Doxorubicin [97]. The gel formation occurs through two steps, self-assembly and then metal complexation. Guanosine monophosphate first self-assembles into highly ordered G-quadruplex structures as seen in Figure 48. These microstructures are then crosslinked through the complexation of the phosphate groups with Fe^3+^ ions (Figure 49), which causes the spontaneous formation of a hydrogel [97]. Further crosslinking of the sugar moieties by Ca^2+^ (Figure 48) was found to increase the mechanical integrity of the gel’s microstructures. The Ca^2+^ cross-linking was also found to increase the hydrogel’s biocompatibility. Rheological studies revealed that the hydrogel exhibited thixotropic properties, allowing for extrusion-based injection [97]. Drug diffusion studies showed a zero-order release of Doxorubicin, a chemotherapeutic, over several days. The drug release was found to be dependent on the pH of the phosphate buffers, with acidic buffers releasing at a faster rate [97]. The hydrogels were found to be stable after 20 days in a pH 7.4 phosphate buffer, with only a 3% release of the loaded drug. This pH sensitive biocompatible metallogel drug delivery system shows potential as a soft material for biomedical applications.

**Figure 50:**
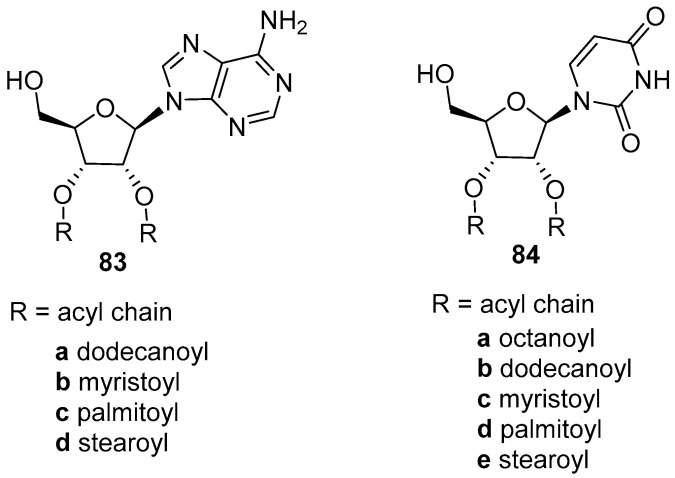
Adenosine nucleolipid derivatives **83a-d** and uridine nucleolipid derivatives **84a–e** [98].

Nuthanakanti et al. designed and synthesized adenosine- and uridine-fatty acid conjugates as shown in Figure 50 [98]. The adenosine-diesters **83a–d** did not form gels in organic solvents. The uridine derivatives with longer acyl chains **84b–d** formed stable gels in several organic solvents. These compounds formed distinct morphologies individually; however, interesting co-assembly was observed when mixed. Mixtures of adenosine and **84c–d** were studied, and their self-sorting or co-assembling properties were analyzed. Increases in mechanical strength were observed in several gel mixtures when compared to gels formed from the individual components [98]. This study showed that mixtures of gelators can be used to tune mechanical strength and construct unique higher ordered microarchitectures.

**Figure 51:**
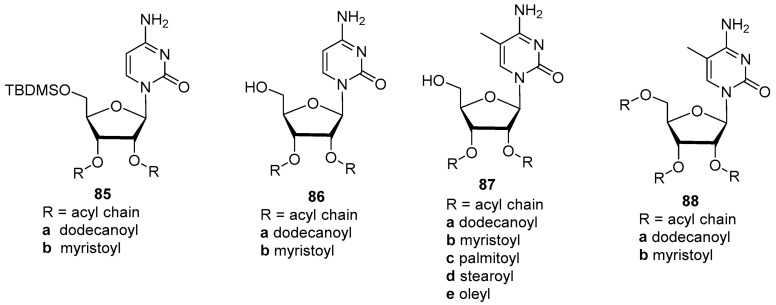
Conjugated cytidine and ribothymidine derivatives [99].

In a related study, cytidine and ribothymidine acyl derivatives were used for the design of supramolecular gels and stimuli-responsive smart materials (Figure 51) [99]. As previously described, the small variations in the chemical structure can result in a wide array of self-assemblies. The self-assemblies occur due to the driving forces such as hydrogen bonding, CH-π, and hydrophobic interactions. As shown in Figure 51, the cytidine diacyl derivatives **86a–b** and the triacyl ribothymidine derivatives **88a–b** did not form gels in the tested organic solvents, however, the diacylated ribothymidines produce stable organogels in various polar and non-polar solvents [99]. The longer acyl chain nucleolipid **87d** was the most efficient organogelator, forming gels at lower MGCs than the others. The DMSO gel of **87d** also showed gel to solution response upon addition of mercury ions.

#### 2.8.3. Nucleotides with Fluorescence Functions

**Figure 52:**
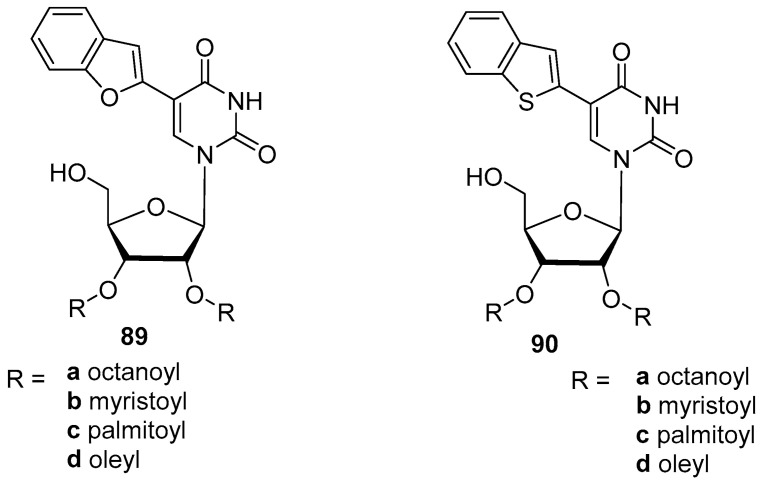
5-Benzofuran and 5-benzothiophene based nucleolipid gelators derived from uracil [100].

Nucleolipid based molecules with inherent fluorescent properties were developed from the modification of 5-Benzofuran and 5-benzothiophene to give compounds **89–90** shown in Figure 52 [100]. Only **89b–c** and **90b–c** were able to form gels in DMSO with relatively low sol-gel transitions temperatures between 32–48 °C and the stability was seen to increase with longer aliphatic chains [100]. All latter DMSO gels were fluorescent when placed under UV light at 365 nm signifying the retention of those properties after self-assembly. Gelator **90c** was selected to investigate the multi-stimuli-responsiveness of the gels and a sol to gel transition was observed for several different processes, heating/cooling and also heating/sonicating. In the presence of bases (DBU) the gel formed a solution, but would reform the gel if acid (trichloroacetic acid) was added [100]. As it relates to metal ions (Hg^2+^, Cd^2+^, Pb^2+^, Ag^2+^ and Zn^2+^), the gel was only responsive to the mercury ion as evidenced by the instability of the gel [100]. On the other hand, several anions (F^−^, HSO_4_^−^, Br^−^, Cl^−^, AcO^−^ and I^−^) were also tested and the gels were stable in the presence of all anions except AcO^−^ and F^−^ [100]. These gelators can be exploited as novel and versatile stimuli-responsive optical materials for a multitude of applications as presented in the paper.

**Figure 53:**
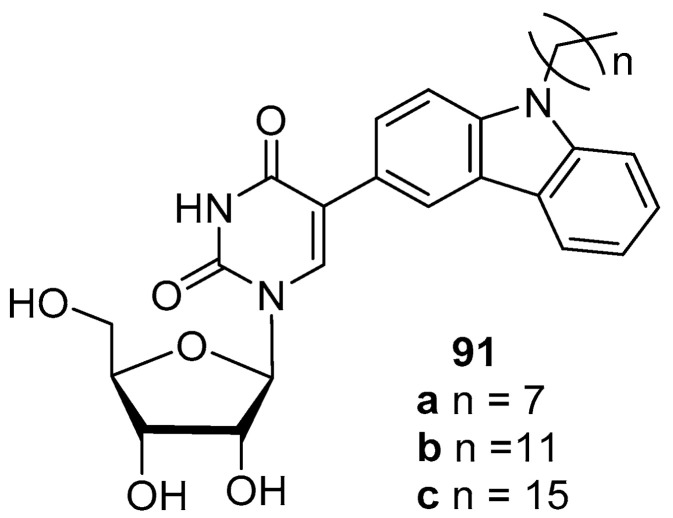
Nucleosides functionalized with carbazole [101].

Another example to show the use of nucleosides as functional organogels was researched by Jia et al. This research showcased luminescent π-gelators. The gelators are soft materials with a wide variety of properties such as intense luminescence, charge carriers, and electronic conductivity [101]. The function of these gelators is commonly used in fluorescence imaging as well as chemo sensors. These biomolecules used as organogels were investigated for a variety of biological applications. Nucleosides have been shown to exhibit good self-assembly directly correlated to hydrogen bonding. A series of new π-gelators shown in Figure 53 below were synthesized by coupling alkyl carbazole with uridine [101]. These fluorescent nucleolipids could form organogels displaying different morphology in solvents such as toluene, and the driving forces for gelation included van der Waals force and hydrogen-bonding [101].

**Figure 54:**
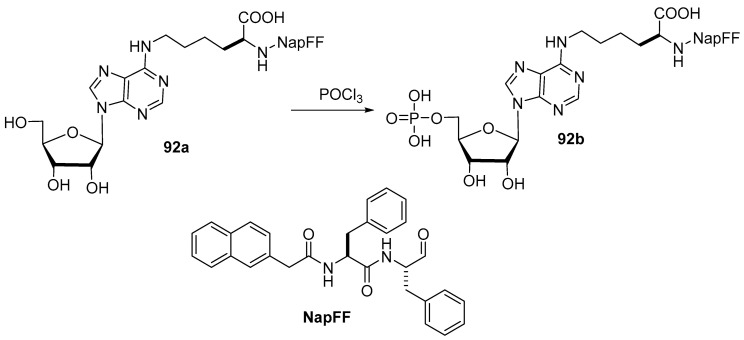
Synthetic route for the adenosine monophosphate-based gelator [102].

Du and his colleagues reported an adenosine-based gelator that was triggered via enzymatic self-assembly (Figure 54). Compound **92a** was produced by adding a known hydrogelator, naphthalene-Phe-Phe-Lys (NapFFK) to adenosine and subsequent treatment with phosphoryl chloride gave the phosphorylated form **92b** [102]. Dephosphorylation of **92b** generates compound **92a** which was able to form a hydrogel at 0.8 wt % and the consecutive TEM images depicted long continuous fibers within the gel matrix [102]. On the other hand, the gelator precursor **92b** does not really form any fibers on its own, so enzymatic action was credited for the self-assembly seen in the gel of **92a** [102]. Their work demonstrated that new biofunctional materials can be obtained by using the enzymatic approach to generate hydrogels from nucleosides like adenosine.

## 3. Introduction to Stimuli-Responsive Gels

There is an increased need for smart materials to be utilized in regenerative medicine to aid in disease treatment. In order to successfully construct stimuli-responsive supramolecular systems, a modular and hybrid molecular design is used in order to introduce artificial chemical reactive groups into biomolecules or bio-related molecules [103]. The main classifications for these materials are physical, chemical, and biological stimuli; with the materials responding to more than one type deemed as multi-stimuli [25]. As it relates to biological stimuli, enzymes are normally the stimuli which have many benefits especially for peptide bond creation as the only other product in most cases is water. The types of physical stimuli that have been comprehensively studied are electrical/magnetic fields, temperature and light [25]. Some stimuli-responsive materials are reversible in terms of their interactions. This special characteristic affords for the sol-gel transition and vice versa which allows for variations in the properties of the gel matrix. Many biocompatible hydrogels that are stimuli-responsive have also been investigated to be applied in immunotherapy as vaccine adjuvants because they are non-immunogenic [25]. Many more applications are being studied for stimuli-responsive materials as there are many challenges and questions that still need to be addressed. The several types of stimuli-responsive gelators are discussed next, these include pH, light, and enzyme responsive gelators.

### 3.1. pH Responsive Gelators

Gelators are structurally unique compounds that can be functionalized for usage in various applications. Within the structural diversity, it is possible to design structures based on external stimuli. pH will be the external stimuli discussed in this section. There are two types of pH-responsive systems, the first type is through protonation and deprotonation [104]. If the structure contains an acid or a base, then with the addition of acid or base it can be positively charged from protonation, negatively charged from deprotonation, or neutral [105]. During the protonation/deprotonation process the gel can be transformed from gel to solution and back to gel upon addition of acid or base. The second class of pH responsive gelators are those with pH labile functional groups incorporated into the structure of the molecules. Therefore, instead of protonation and deprotonation the molecule will break apart into different classes of functional groups [106]. To further understand this phenomenon, we will discuss in detail a few examples in the recent literature.

In a recent study, cytidine-5′-monophosphate (5′-CMP) mediated akaganeite (ß-FeOOH) based nanoparticles were prepared and self-assembling hydrogels were obtained [107]. At low pH, the nanoparticles remained as a colloidal solution, but can be converted to hydrogels at pH 5.5–7 at 35 °C or pH 7–8.5 at 40 °C. The nanohybrid framework gave porous hydrogels whose self-assembling mechanism is attributed to the change in confirmation of the ribose sugar from C2′-endo to C3′-endo which boosted the non-covalent interactions [107]. The gels exhibited low cytotoxicity with self-healing and injectable characteristics confirming their biocompatibility [107]. Fairly high values in the storage modulus were obtained from the rheology data which shows these gels have strong mechanical properties. As it relates to applications, the research demonstrated that these gels could effectively load and release drugs at physiological pH and have potentials for several biomedical applications.

**Figure 55:**
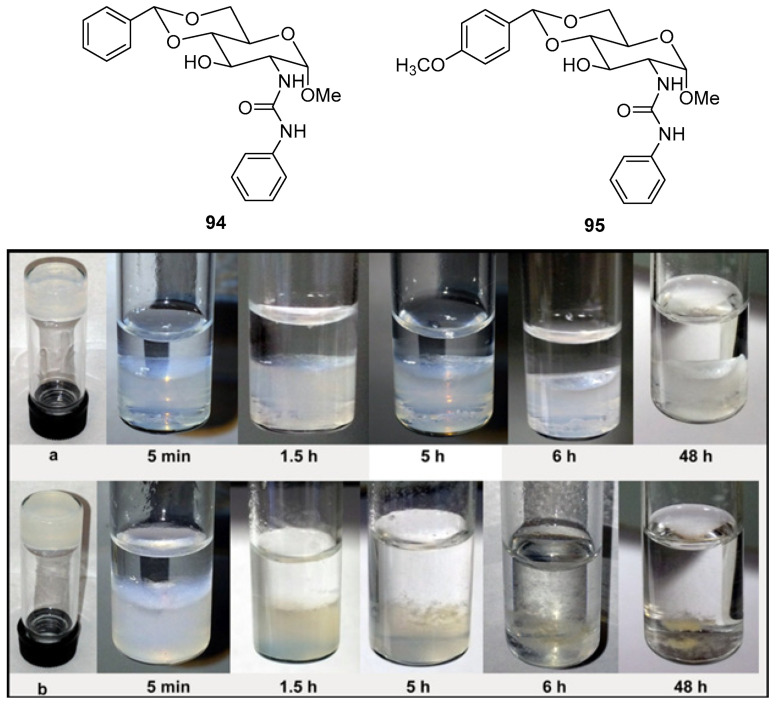
Photographs of respective gels in acidic conditions. (**a**) A gel formed by compound **94** in DMSO/H_2_O (1:2) at 5 mg/mL. (**b**) A gel formed by compound **95** in DMSO/H_2_O (1:2) at 5 mg/mL. Reprinted with permission from [35]. Copyright 2014 Beilstein-Institute.

A few carbohydrate-based gelators prepared in the Wang group were also pH-responsive. These include a series of D-glucosamine gelators which formed several stimuli-responsive gels as depicted in Figure 55. The benzylidene acetal protection in **94** was replaced with a p-methoxybenzylidene acetal protection in **95**, making the protecting group more acid labile. As seen in the image in Figure 55, the gels made by compounds **94** and **95** were exposed to 1 mL of sulfuric acid solution (pH 1) at different time periods, and the photos show the steady degradation of the gels overtime [35]. After 48 h, compound **94** was not fully dissolved but compound **95** was almost fully dissolved [35]. These stimuli-responsive gels can be utilized for the rapid release of drugs in an acidic environment [35].

### 3.2. Photosensitive Gelators

Besides acid and base sensitive gelators, light can be an effective stimulus to form stimuli-responsive gelators. Photo-responsive gelators can have a variety of applications due to the interesting light controlled phase transition [108]. Photoresponsive functional groups such as azobenzene have been used with carbohydrate moieties to afford interesting light sensitive gelators [109,110]. Glycoconjugates with fluorescent properties have also been reviewed [25].

#### 3.2.1. Photo-Isomerizable Gelators

**Figure 56:**
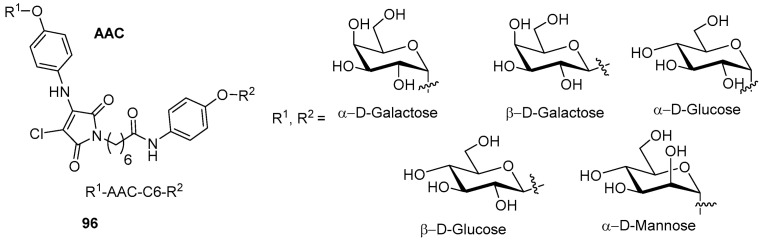
Structures of N-alkyl-2-anilino-3-chloromaleimide (AAC) containing bola-amphiphilic glycolipids with peripheral sugar units [111].

Stimuli-responsiveness is not only limited to the gel to solution transitions. Oosumi et.al. have designed and synthesized N-alkyl-2-anilino-3-chloromaleimide (AAC) containing bola-amphiphilic glycolipid LMWGs, which exhibit reversible thermochromism in addition to reversible gel-sol transitions [111]. These bola-amphiphiles contain either homo- or hetero-saccharides at their peripheries, linked through the AAC moiety and a hydrophobic core (Figure 56) [111]. Upon heating to 45 °C, well below T_gel_, the gel appearance changed from opaque to transparent, indicating a change in aggregation state without a complete disassembly of the gel scaffold [111]. When studying this phenomenon, a red shift was observed on UV-vis. Additionally, the differential spectrum showed that the gel to solution transition was accompanied by an increase in absorbance at 445 nm and a decrease at 383 nm, verifying a color change with the self-assembled state [111]. This work is further being explored in bio-applications as sensing materials for glycol-related enzymes.

**Figure 57:**
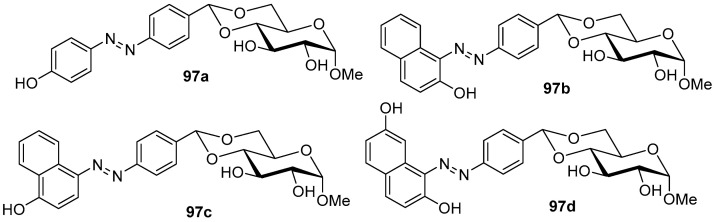
Structures of sugar azobenzene-derived LMWGs [112].

Multi-stimuli-responsive supramolecular gelators (MRSGs) can be rationally designed by functionalizing gelators with stimuli-responsive functional groups. As shown in Figure 57, Khayat et al. designed and synthesized glucose-based MRSGs containing aryl azo moieties for photo-responsiveness and hydroxyl groups as pH and metal ion responsive groups [112]. The gels formed by the sugar azobenzene derivatives were found to collapse within 30 min of exposure to 254 nm and 360 nm UV light. Additionally, full transformation to a solution state was found to occur with prolonged exposure to daylight (2–4 days). At a pH higher than 12.4 and lower than 1.6, gels were found to transition to the solution state [112]. Gels formed by the mono-hydroxyl naphthyl derivative showed a selective response to Cu^2+^ ions among an assortment of metal ions.

**Figure 58:**
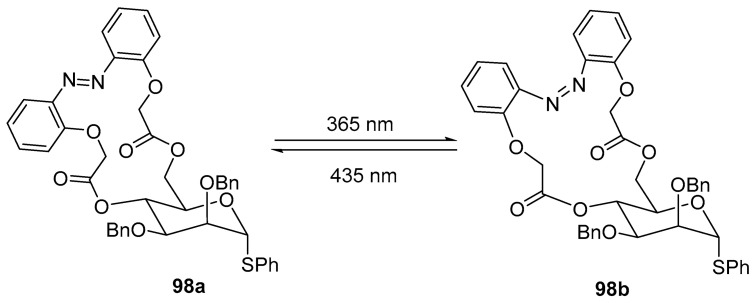
The cis-trans isomerization of the macrocyclic compound [113].

Lin et al. reported an azobenzene carbohydrate-based macrocycle LMWG **98a** which undergoes cis-trans isomerization when irradiated with specific wavelengths of light [113]. The azo moiety in the macrocycle is converted to the cis isomer after treatment with 365 nm light and reverts to the trans-isomer with 435 nm irradiation (Figure 58). Switching cycles were repeated over 30 times, showing excellent fatigue resistance [113]. The Z isomer was found to be very stable, with a half-life of 51 days. Cyclohexane gels formed by the macrocycle were found to undergo an irreversible gel to solution transition upon irradiation with 365 nm light. The results of this study show how novel intelligent photo responsive sugar based macrocyclic LMWGs can be rationally designed.

**Figure 59:**
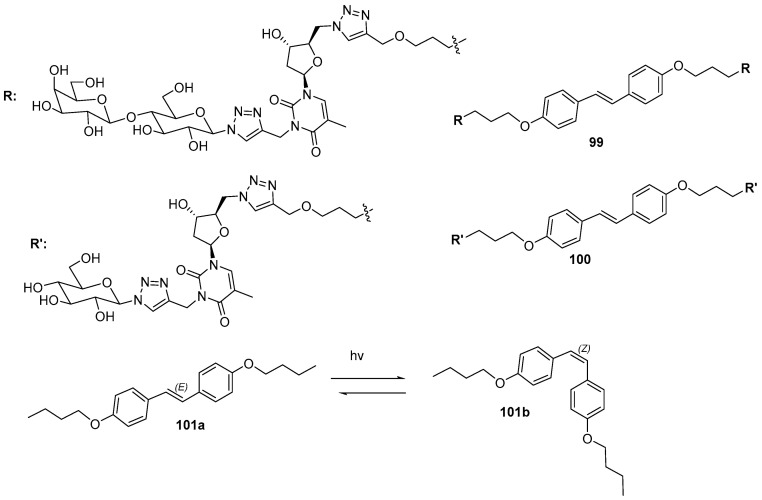
Structures of light-sensitive glycosylated nucleoside-based bolaamphiphiles (GNBAs) **99–100** [114].

In addition to azo functional groups, stilbene moieties can be incorporated into LMWG designs to create photosensitive smart materials. Baillet et al. designed and synthesized a series of stilbene containing glyconucleoside bolaamphiphilic LMWGs [114]. The compounds **99** and **100** displayed gelation properties in ethanol–water mixtures (Figure 59). The gels formed by these compounds also showed a gel–sol transition under UV irradiation due to the E–Z isomerization of the stilbene unit [114]. Upon radiation with 312 nm UV light, UV spectra indicated that the Z-isomer was mainly present in solution, indicating that the cis-trans isomerization destroyed the supramolecular architectures of the gel [114]. These multi-stimuli-responsive soft materials have plausible applications in replacing the current polymer-based UV controlled drug delivery systems due to their faster response time to UV light.

#### 3.2.2. Diacetylene Containing Photoreactive Gelators

Among photoresponsive gelators, diacetylene derivatives can be considered as photo-reactive gelators if the gelator can photopolymerize. Polydiacetylenes are interesting polymers exhibiting color transitions in response to environmental changes and binding to biomolecules; they also have non-linear optical properties and show other important optical effects. The typically blue to red color change of polydiacetylenes makes them useful candidates for sensing and the preparation of stimuli-responsive materials. The color transition properties of the polydiacetylene gels can be used to predict environmental condition change, such as in the event of binding to a biological agent.

**Figure 60:**
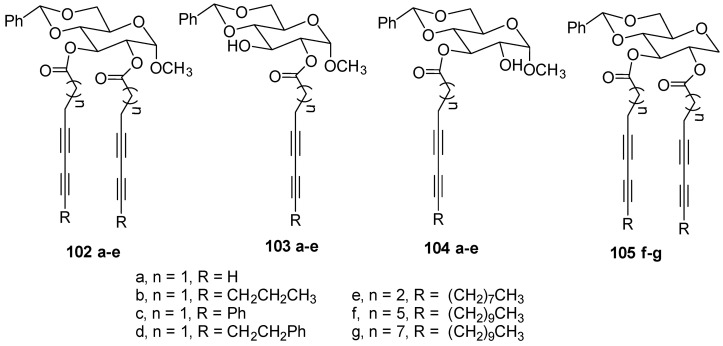
Structures of diacetylene containing glycolipids from D-glucose [30,31], n is the number of repeating CH_2_.

**Figure 61:**
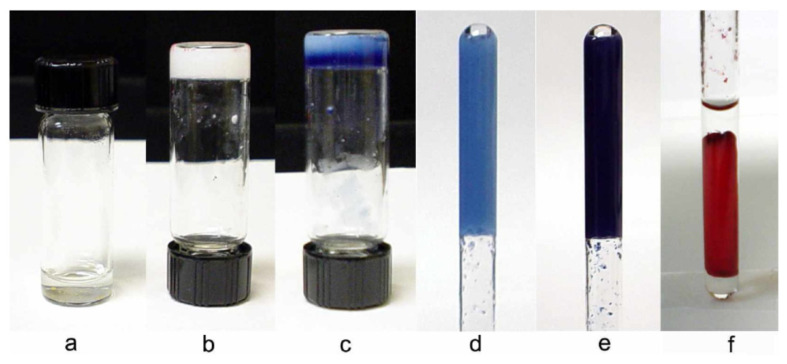
The gel formed by compound **105f** in ethanol at below10 mg/mL. (**a**) a clear solution of the heated gelator, (**b**) a stable gel after cooling to room temperature, (**c**) the gel turned blue after irradiation of UV lamp from the top of the glass vial, (**d**) the gel was transferred to a quartz tube and irradiated for 1 min, (**e**) the same gel turned dark blue after UV treatment for 3 min, (**f**) the red gel was obtained by heating the gel in (**e**). Reprinted with permission from [31]. Copyright 2011 Beilstein-Institut.

**Figure 62:**
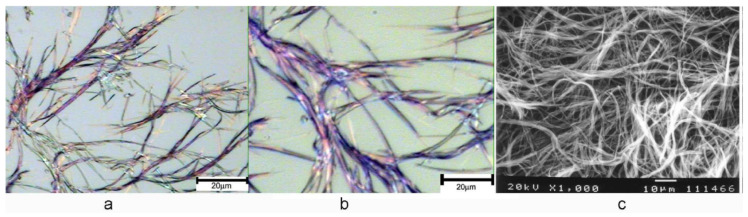
Optical micrographs of the ethanol gel in Figure 61 by compound **105f** under bright field (**a**,**b**) and scanning electron micrograph (**c**). These gels were prepared by transferring the ethanol gel after UV treatment for 3 min. Reprinted with permission from [31]. Copyright 2011 Beilstein-Institut.

Carbohydrates can also be utilized to form polymeric gelators by the incorporation of diacetylene groups on respective sugar molecules. The creation of gelators that comprise of diacetylene are rather interesting and fascinating because they can respond to multiple stimuli and have different color transitions as well as gel-sol transitions, which allows for their usage in the design of sensors [30]. The Wang group has discovered that a variety of diacetylene containing glycolipids are effective gelators for organic solvents including ethanol, and ethanol/water mixtures [30,31]. The systematic study of the effect of chain length on gelation properties of a series of diacetylene containing glucose derivatives was carried out. The structures of these glycolipids are shown in Figure 60. Cross-linking of the polymerizable groups in the gels can result in new materials with desirable properties. The gels formed by these diacetylene compounds can be polymerized and showed the typical blue to red color transition upon heating. An example of a polymerized diacetylene containing gelator in ethanol is shown in Figure 61 (**a–f**) [31]. The gels were characterized by obtaining scanning electron micrographs (SEMs) and optical micrographs (OMs). One example is shown in Figure 62 [31]. Optical microscopy studies showed that the molecules form interesting architectures such as tubules, rods, sheets, and belts. Scanning electron micrographs of the gels showed fibrous assemblies.

Even though compounds **105f and 105g** were less effective gelators, they were able to polymerize when exposed to UV-light which proves they are stimuli-responsive and have the potential to be utilized in applications that require the latter phenomenon [30,31].

**Figure 63:**
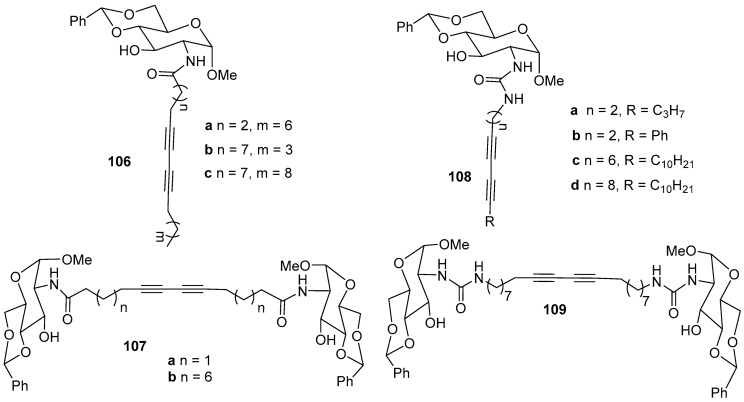
Structures of diacetylene containing amide and urea derivatives [38], n and m represent the number of repeating CH_2_ unit.

The Wang group also incorporated amide and urea functional groups and synthesized ten different derivatives as shown in Figure 63. These compounds were found to be efficient gelators for a series of organic solvents. Gelator **106c** was able to form a gel in isopropanol ethanol and toluene; its lowest concentration was in ethanol at 0.8 mg/mL [38]. Compound **107b** was also able to gel in the above mentioned solvents but also in EtOH:H_2_O (*v*/*v* 1:2) with a concentration of 2.5 mg/mL [38]. The molecules were polymerizable and depicted color transitions under UV-light and may be useful as sensors to identify changes within an environment [38].

**Figure 64:**
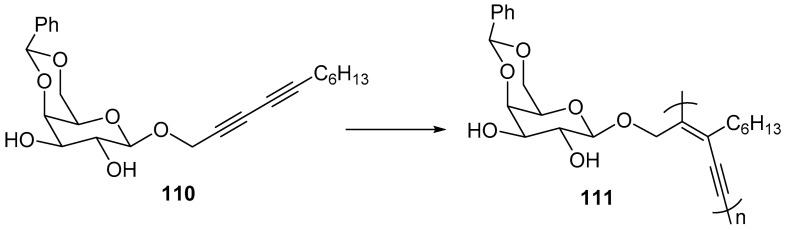
Diacetylene and glyco-polymer-based gelators [115], n represents the number of repeating units.

Krishnan et al. reported diacetylene-based gelators from a previously reported 4,6-O-benzylidene methyl β-_D_-glucopyranoside platform [115]. Compound **110** formed gels in oils and non-polar solvents at low concentrations (Figure 64) [115]. In the gel phase, the molecule was able to align itself such that the diyne groups were close enough to each other to polymerize when exposed to UV light at a wavelength of 300 nm over a 2 day period [115]. The glyco-polymer **111** that resulted from the polymerization reaction was then utilized for lectin binding studies with Ricinus communis lectin (Rcl), peanut agglutinin (Pna) and Erythrina crista-galli lectin (Ecl) that were labeled with fluorescein [115]. The glyco-polymer had a very high affinity for the galactose-binding lectins, 1000 times higher than the monosaccharide counterparts that were employed in the test [115]. These gels show very good results for the advancement of bio-based materials for applications in lectin binding.

### 3.3. Enzymatic Responsive Gelators and Their Biomedical Applications

The process of gelation can be triggered using external stimuli; therefore, in the recent decade, many researchers have been studying the ability for self-assembly and gelation to be enzymatically controlled [25,115]. Enzymes are biological catalysts that can transform or form new bonds in molecules under physiological conditions [116]. Enzymes can be used to trigger gelation or for breaking down the gel to aid in the controlled delivery of drugs or biomolecules [117]. In the presence of enzymes, many gelators do not have the stability to withstand the enzymatic degradation if it has the substrate moiety that the enzyme acts on. There are many benefits of using enzymes as stimuli over other types of stimuli; the reactions of enzymes are more selective and specific, they require moderate conditions for reacting and are highly efficient, also they do not require assistance from other stimuli to act because they are endogenous [117]. The common enzymes that have been investigated in the literature are phosphatases, oxidoreductases, proteases, kinases and esterases [117]. Enzyme responsive materials have quite interesting properties that allow for them to be utilized in many biomedical applications [117]. Whilst many peptides and polymers have been exploited as enzyme-triggered systems, not many carbohydrate-based materials have been reported in the literature and much research is needed in the latter area. In this section of the review, we hope to highlight a few examples of sugar-based soft materials that are responsive to enzymes and how they were designed.

**Figure 65:**
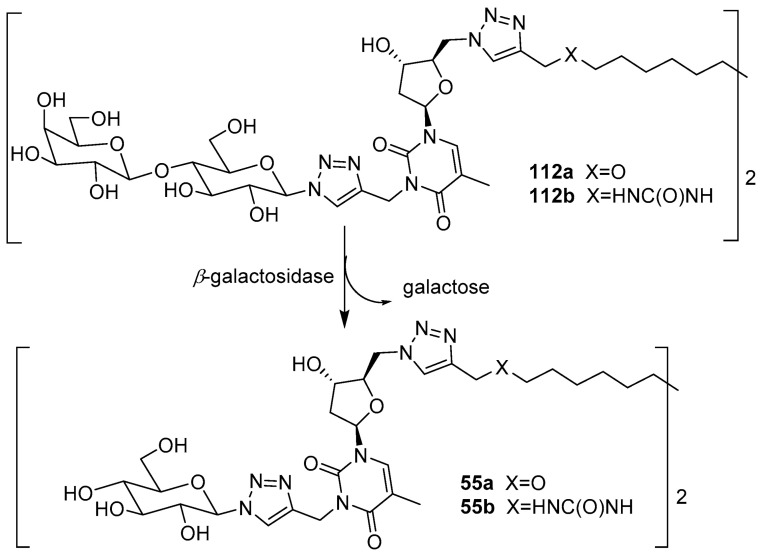
Structures of glyconucleo-bolaamphiphiles [118].

Many supramolecular assemblies can be triggered by enzymatic stimuli and one enzyme that is utilized heavily in the area is β-galactosidase (β-gal). Stimuli-responsive materials were generated using glyconucleo-bolaamphiphile structures (GNBAs) [118]. The glucosyl triazole derivatives were reported as gelators [84] and the authors envisioned that preparing the lactose precursors of these compounds would result in lactase responsive pro-gelators. Therefore, lactose-based gelator precursors **112a–b** were synthesized using click chemistry to attach the sugar component to the nucleotide (Figure 65) [118]. At a concentration of 5% *w*/*v* in PBS, **112a** was able to form a viscous solution but **112b** actually formed a hydrogel at 2% *w*/*v* [118]. Upon being treated with β-gal, both compounds formed hydrogels at different concentrations and the rheology data suggested the supramolecular assemblies after enzymatic reactions had stronger mechanical properties than their precursors [118]. Their results demonstrate that galactosidase responsive materials can be utilized to change several functions within a cell to investigate more therapeutic methods [118].

**Figure 66:**
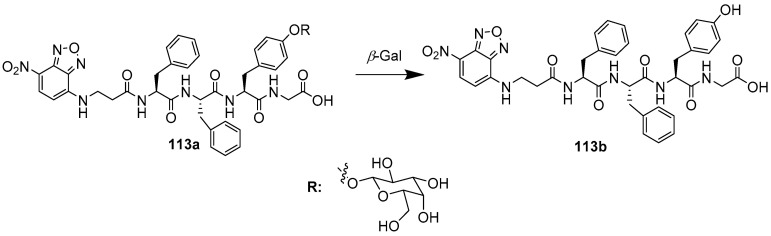
Gelator precursor for β-galactosidase [119].

There are other reports of supramolecular assembly resulting from enzymatic cleavage in the presence of a sugar hydrolase, β-galactosidase (β-Gal). In another example, the molecular design was in the form of diphenylalanine (FF) and tyrosine attached to 4-nitro-2,1,3-benzoxadiazole (NBD) along with a β-galactose sugar moiety [119]. The sugar component was attached directly to the tyrosine residue to increase the solubility of the gelator precursor **113a** so that β-Gal could cleave it and hence result in self-assembly [119]. The structure of the gelator precursor can be seen in Figure 66. The conditions for enzymatic cleavage were at 37 °C in phosphate-buffered saline resulting in the formation of a brown gel at 0.5 wt %. A fibrous morphology was observed under the transmission electron microscope [119]. Subsequently, the gelator precursor was incubated with senescent HeLa cells and their results demonstrated that the compound could self-assemble within the cells and has the potential to be utilized for detecting or pinpointing these types of cells [119]. They also evaluated if this intracellular self-assembly could help in removing endothelial senescent cells. This was done by treating human umbilical vein endothelial cells (HUVECs) with conditions that would cause them to become senescent and then the resulting cells were then treated with the gelator precursor [119]. Their findings demonstrated that the compound had significant toxic effects on the cells and 70% of the cells were eradicated at a concentration of 400 μL for the gelator precursor [119]. The researchers were also able to confirm that cell death was from the intracellular self-assembly of the precursor due to the cleavage of the galactose sugar component in the presence of β-Gal [119]. Overall, the article presented a molecule that could be used for selectively discovering and destroying senescent cells by β-Gal instructed intracellular self-assembly.

**Figure 67:**
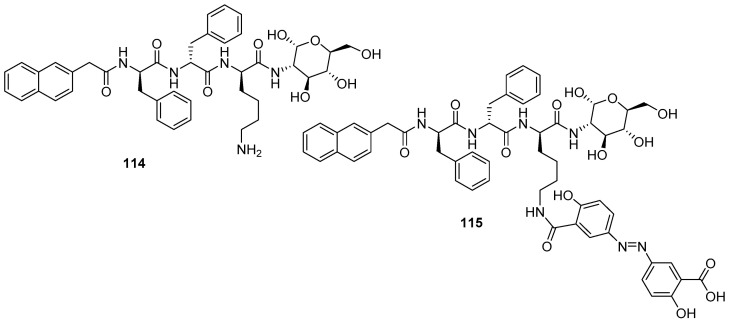
Structure of glycoconjugates **114** and **115**.

An interesting example of enzymes acting as a stimulus was reported by Xu’s group; examples of these molecules are shown in Figure 67. The glucosamine peptide based gelator **114** formed biocompatible hydrogels [120]. The derivatives of **114** were covalently attached to olsalazine an anti-inflammatory drug, to give compound **115**. The molecules were able to self-assemble with the drug attached to form nanofibers in an acidic environment and the studies showed that after being treated with azoreductase, the drug mesalazine was released from the gel [120]. In addition to that, the hydrogel was able to mimic mucus and aid in restoring the injured mucosal layer. The supramolecular assemblies of several other glycopeptides were further studied for applications in cell functions [121]. In another related study, enzymatic responsive gelation was reported using HINT-1 (Histidine triad nucleotide-binding protein 1). The nucleoside phosphoramidate had various bases from purines to pyrimidines and comprised of a naphthyl group at the N-terminal of the azido-labelled peptide [122]. In the presence of the HINT 1 enzyme, well defined fibers formed extensively to give hydrogels.

**Figure 68:**
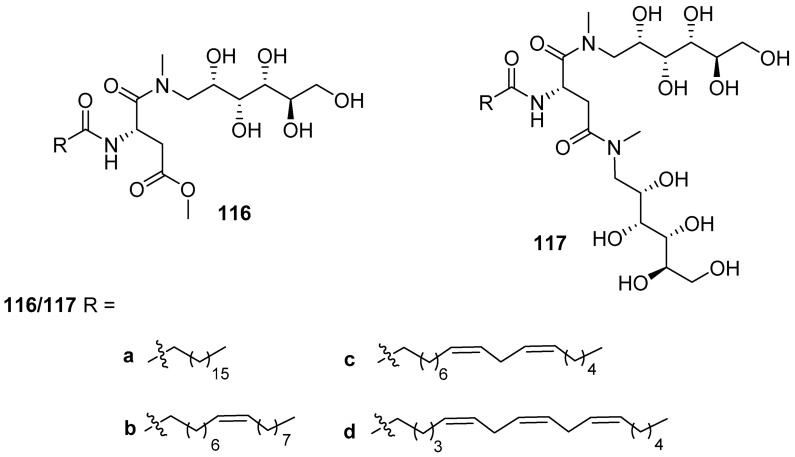
Structures of eight glycolipids containing aliphatic tails with increasing degree of unsaturation [123].

Sekhar et al. synthesized a series of eight glycolipids containing either one or two polar N-methyl-D-glucamine headgroups and one or two aliphatic tails with increasing degrees of unsaturation as shown in Figure 68 [123]. Glycolipids with small polar head groups and less unsaturated aliphatic tails, **116a** and **116b**, formed hydrogels, while the glycolipids with the higher unsaturated aliphatic tails **116c**, **116d**, **117c** and **117d** exhibited water-induced organogelation through agitation. Increasing the size of the polar head group was found to increase the glycolipids’ ability to form hydrogels. The gels formed from these glycolipids were found to be capable of encapsulating both hydrophobic and hydrophilic bioactive molecules such as curcumin and riboflavin. The capacity of the gels to encapsulate these molecules increased with larger sized polar head groups. The enzymatic triggered release of bioactive molecules was studied using amidase-like trypsin. Hydrolysis of the glycolipid led to the degradation of the gelator and thus the release of the bioactive molecules. The hydrogels were also found to be capable of removing chromium and copper from vegetable oil and the removal capacity was found to increase with the unsaturation of the hydrophobic tail [123]. The gels formed from these glycolipids may have applications in metal removal for the purification of vegetable oils and in gel-based delivery systems.

**Figure 69:**
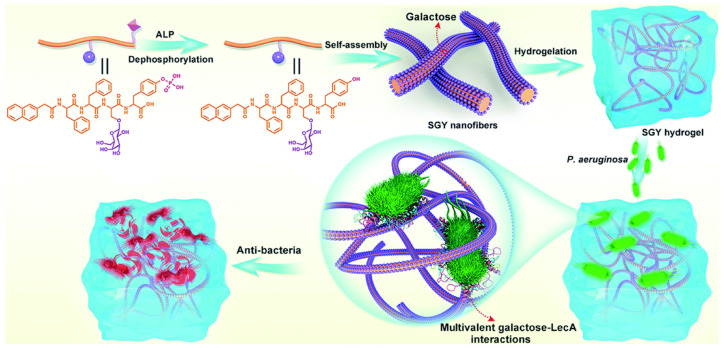
Enzyme instructed self-assembly and antibacterial properties of a galactose-based gelator. Reprinted with permission from [124]. Copyright 2020 Royal Society of Chemistry under the creative commons license.

The most common enzyme that has been utilized as a trigger in sugar-based gels is alkaline phosphatase (ALP). The first gelator example described here was designed with a peptide segment containing a phosphate group that can be cleaved by ALP, a naphthyl group for assisting with self-assembly through aromatic interactions and the galactose sugar headgroup for interacting with lectins on *Pseudomonas aeruginosa* to investigate its antibacterial properties [124]. A schematic diagram detailing the findings of the research is shown in Figure 69. The molecular self-assembly happened after it was dephosphorylated and the fibers formed in such a way that the galactose moiety was on the outside of the self-assembled fibers, allowing for it to interact with the lectin on the bacterial cell wall [124]. The findings demonstrated that the multivalent galactose clusters within the hydrogel network were able to bind galactose-binding proteins such as Lec-A that are specific to *P. aeruginosa* [124]. This resulted in the inhibition of microbial growth and biofilm formation [124]. Therefore, in this case, the enzyme was utilized as a trigger for self-assembly which formed a gel that later acted as an antibacterial agent.

**Figure 70:**
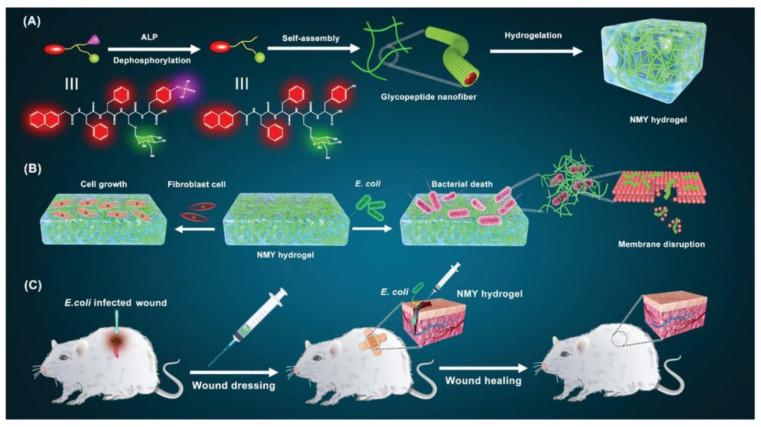
Enzyme instructed self-assembly of a hydrogelator and its applications. (**a**) Enzyme instructed self-assembly of the glycopeptide gelator forming a fibrous network within the hydrogel; (**b**) Impact of the hydrogel on the growth of fibroblast cells and *E. coli*; (**c**) Prospective usage of the hydrogel for wound healing applications in a mouse model. Reprinted with permission from [125]. Copyright 2019 Jon Wiley and Sons, Inc.

Two other captivating examples were reported in the literature utilizing the same design principles of a molecule with a naphthyl group attached to a tetrapeptide unit containing a phosphate group, incorporating ALP as the enzymatic stimuli for self-assembly and a sugar moiety for lectin binding. A glycopeptide-based gelator composed of D-mannose and Phe-Phe-Ser-Tyr(H_2_PO_3_) was reported as an antibacterial agent as well as for wound healing applications [125]. As shown in Figure 70, section A, the mannose was specifically included to enable mannose-lectin interactions that would bring about protein agglutination of the lectin Concanavalin A (ConA), disturbance of the cell membrane and bacterial adhesion [125]. The hydrogel formed at 1 wt % after the phosphate group was cleaved in the presence of ALP and it had the typical fibrous morphology. The antibacterial properties were tested using cultures of *Escherichia coli*; they employed strain ATCC-25922 which has a mannose-binding protein FimH adhesion incorporated in its cellular model [125]. Upon incubating the bacteria with the hydrogel, visible bactericidal activity was observed and it increased as the concentration of the hydrogel increased, as depicted in Figure 70, section B [125]. To investigate the specificity of the hydrogel to mannose-binding proteins, *Staphylococcus aureus* was also tested as it does not contain these types of proteins that would form interactions with mannose [125]. The results illustrated that the gel had no effect on *S. aureus* which further confirms their hypothesis. Based on the rheological studies, the hydrogel had thixotropic properties that allow the gel to be injected. Cell viability assays confirmed that it is capable of being employed as a medium for the growth and proliferation of mouse fibroblast cells (NIH-3T3) [125]. Further in vivo studies revealed that the hydrogel can be successfully utilized as a substance to dress wounds that would protect the wound from bacterial infections and allow for proper regeneration of skin at a fast enough rate [125].

## 4. Applications of Sugar-Based LMWGs

### 4.1. Environmental Remediation

The treatment of wastewater involving harmful chemical and oil spills remains a huge challenge in our society. In the early days, several synthetic materials were utilized in the process of cleaning up pollutants, but some of those substances were even more toxic than the pollutants they were removing [126]. In the quest to use a more environmentally friendly approach, many researchers have turned to soft materials such as gels that are non-toxic and biodegradable to aid in solving this worldwide problem [126]. Gels have attracted much attention for this application because they operate at a wide pH range and have a large surface area for the absorption of pollutants [127]. In addition to that, the gelator molecules can be easily modified to generate new molecules with stable and strong mechanical properties [127]. Several classes of pollutants that have been studied for their removal include inorganic ions, petrochemicals, dyes and other pharmaceuticals [127]. In order to design successful candidates for wastewater treatment, one must ensure that the gelator has chemical functionalities that allow for it to have increased interactions with the particular pollutant. However, due to contradicting properties of both substances involved the construction of materials remains a huge challenge. Nevertheless, researchers have had some success in generating very robust materials that can be reused and aid in the sustainable removal of contaminants from water [126]. This section of the review will highlight several carbohydrate-based materials that have been developed in the past couple of years that were successful in the removal of a variety of wastes from water.

**Figure 71:**
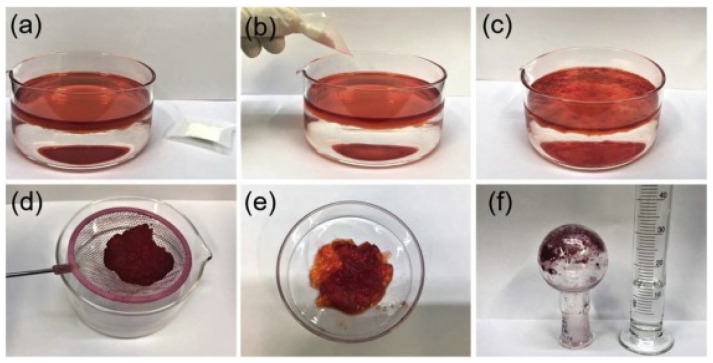
Process of solidifying and removing toluene from an oil-water mixture: (**a**) Aqueous biphasic mixture of toluene, dyed with Disperse Red 152; (**b**) Addition of the powdered gelator; (**c**) Instantaneous gelation of the toluene layer; (**d**) Removal of the solidified toluene layer; (**e**) Toluene gel after removal; (**f**) Graduated cylinder containing the recovered toluene and recovered gelator (separated by vacuum distillation). Reprinted with permission from [72]. Copyright (2020) Elsevier.

Fang et al. designed and synthesized two D-gluconic acetal-based phase selective organogelators. Compound **35b** (Figure 25) was found to solidify toluene, benzene, and o-xylene at room temperature in biphasic aqueous solutions within 10 min of the addition in powder form. High recovery rates of about 82% were achieved for the aromatic solvents, as well as gelator recovery, via distillation [71]. D-Gluconamide derivatives with various aliphatic chains were also shown to be phase selective gelators for oils [72]. Compound **36a** (Figure 26) containing a cis-double bond, was found to create a porous amorphous powder capable of phase-selective gelation of aromatic solvents, refined petroleum products and crude oils. Figure 71 shows the selective removal of toluene from an oil-water mixture by the powder of compound **36a**. Gelation occurred at room temperature instantaneously. This phase selective organogelator showed high recovery rates and is applicable in a wide range of oils and aromatic solvents. The introduction of the cis double bond, which created an irregular structure in the alkyl chain, is most likely responsible for this compound’s ability to gelate solvents at room temperature, when added in solid form.

An arabinopyranoside-based gelator (compound **27b** in Figure 19) was used for the phase selective gelation of crude oil and removal of oil from sea water. They showed that the gelator **27b** with starch composite (1:1, 500 mg) was able to solidify crude oil layer at room temperature instantly, therefore the crude oil powder can be scooped away from the water mixture. The combination of the gelator and starch causes a uniform dispersion of the gelator throughout the oil layer, leading to fast and uniform gel formation. The starch acts as a solid support for gelator dispersal and minimum gelation concentrations as low as 0.34% *w*/*v* were achieved using this method [62]. The results of this study are a significant step towards using LMWGs in solid form for congealing crude oil from biphasic oil-water mixtures.

Hou et al. examined dibenzylidene acetal protected sorbitol compound **37a** depicted in Figure 27, as a LMWG in applications of oil solidification and removal. A wide variety of oils were tested and minimum gelation concentrations of 0.04–0.10 g/mL were observed. Gelation was found to occur within 17 min of adding the powdered gelator to the oils [128]. In further studies, they found that the amount of water used in the experiment did not impact gel formation. The results of this study show that dibenzylidene acetal protected sorbitol is an efficient LMWG in oils and has potential for use in marine oil spill cleanup applications.

**Figure 72:**
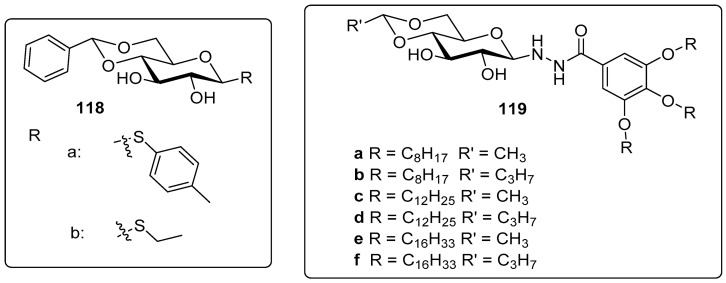
Structure of D-glucose acetal-based gelators **118** and **119** [129,130].

Sureshan et al. designed and synthesized five glucose-derived phase selective organogelators, which can form gels at room temperature when applied as a fine powder. To reduce the water solubility, thioalkyl or thioaryl motifs were installed at the anomeric position of the sugar head group [129]. The resulting compounds were found to selectively congeal oils in aqueous biphasic oil solutions. Gelators **118a** and **118b** (Figure 72) were found to be supergelators in crude oil with minimum gelation concentrations of 0.5 wt % and 0.6 wt % [129]. Compound **118a** was used to solidify crude oil in an oil-water mixture, so it could be scooped out and removed easily.

Soundarajan et al. designed and synthesized a series of 3,4,5-tri-O-benzohydrazide based N-glycosylamine derivatives and explored their use as LMWGs in environmental remediation applications. Each sugar-based gelator in Figure 72 was found to be capable of forming gels in toluene, dichlorobenzene, benzene, mineral oil, coconut oil, petrol, kerosene and crude oil [130]. Benzene could be recovered and removed by the gel from a water-benzene mixture [130]. After distilling the benzene from the gel, the solid gelator was also recovered, showing the recyclability of the gelator [130]. These supramolecular organogels also successfully removed rhodamine B and acid orange from aqueous solutions, showing the potential use as supramolecular sponges for dye removal. This study shows the potential that supramolecular gels formed from carbohydrate based LMWGs have in various environmental remediation applications.

**Figure 73:**
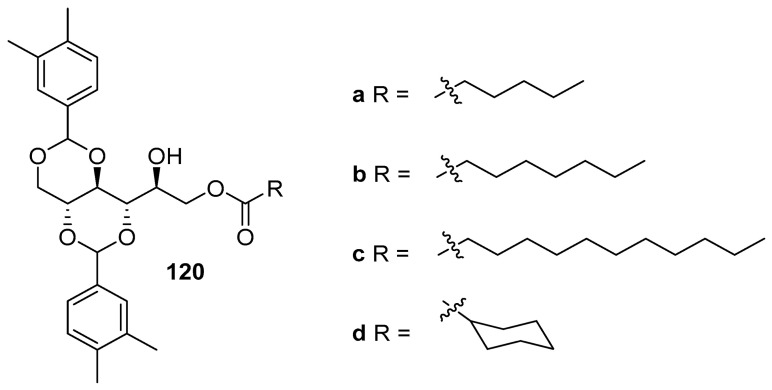
Sorbitol based supramolecular gelator [131].

Song et al. designed and synthesized di-2,3-methylbenzyledine acetal protected sorbitol (DMDBS) derivatives with aliphatic groups of varying chain lengths as supramolecular organogelators (Figure 73) [131]. All DMDBS derivatives were found to form gels in various non-polar organic solvents and oils. Rheological experiments and self-healing experiments also showed that most of the gels possess self-healing properties. Compounds **120a-c** were found to be phase selective gelators, gelling only the organic phase in aqueous biphasic mixtures. Gelators with longer alkyl chain lengths were found to have lower biphasic critical gelation concentrations (BCGC), so compound **120c** was chosen for the experimental recovery of gasoline from an aqueous biphasic mixture. Using THF as a carrier solvent, compound **120c** was able to gel the gasoline layer within 3 min of addition. The solidified gasoline layer was then scooped out of the biphasic mixture and distillation was used to separate the gasoline from the gelator, so the gasoline could be recovered and the gelator could be recycled.

**Figure 74:**
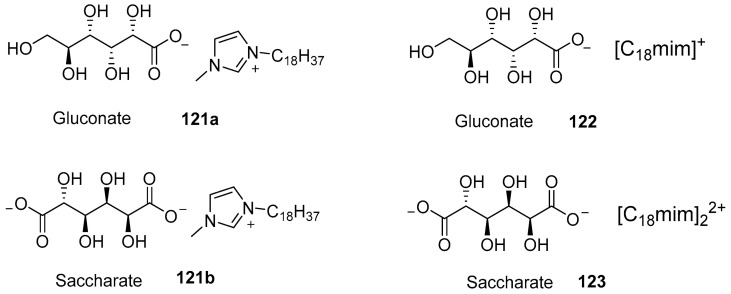
Carbohydrate salt-based derivatives [132].

Rizzo et al. designed and synthesized imidazolium salt carbohydrate derivatives as supramolecular ionic liquid gelators (Figure 74) [132]. The imidazolium cations contain long alkyl chains, making them the component that contributes to aromatic–aromatic interactions as well as the hydrophobic interactions. The carbohydrate-derived anionic components contribute to hydrogen bonding interactions. Gelation was tested in organic solvents, aqueous solutions, and ionic liquids. The [C_18_mim]Br salts were found to be very versatile gelators in water, salt solutions and diesel fuel, while the carbohydrate salts, on the other hand, required solutions with a higher ionic strength. The ionic liquids used for testing were 1-methyl-3-butyl imidazolium [bmim^+^] with either hexafluorophosphate [PF_6_^−^] or N, N-bis(trifluoromethanesulfonyl)imide [NTf_2_]^−^. Applications in water treatment were analyzed, particularly in metal removal. All gels tested were found to remove 60% of chromium within 3 h of exposure. When small samples of these hydrogels were immersed in large chromium solutions, extremely high adsorption capacities were observed. The carbohydrate-derived gels showed better adsorption of chromium compared to the bromide gels. The [C_18_mim]_2_[saccharate] (**123**) hydrogels achieved total adsorption within 24 h and interestingly, were also able to reduce the adsorbed chromium (IV) to the less toxic chromium (III) species [132]. ^1^H NMR experiments using saccharic acid potassium salt and potassium dichromate in D_2_O were carried out to show the plausibility of reduction occurring in the [C_18_mim]_2_[saccharate] hydrogels. This adsorption/reduction property of the imidazolium salt carbohydrate hydrogel can have a significant advantage in water treatment.

Liu et al., previously mentioned in this review, also showed that their gelators have plausible applications in contaminant removal from aqueous solutions, oil spill recovery and in optical devices. The n-butyl acetate xerogel of compound **33c** in Figure 24 was found to be capable of successfully adsorbing iodine and crystal violet from aqueous solutions. After the xerogel was submerged in the solution, almost all of the crystal violet was found to be absorbed into the gel within 24 h as confirmed by UV-vis spectroscopy [69]. Biphasic oil solidification at room temperature was studied by using THF as a carrier solvent. Gelation of the oil layer was found to be instantaneous showing the plausible applications in cleaning up oil spills. The stability, moldability, and transparent nature of these novel soft materials make the ideal candidates in the development of flexible optical devices.

**Figure 75:**
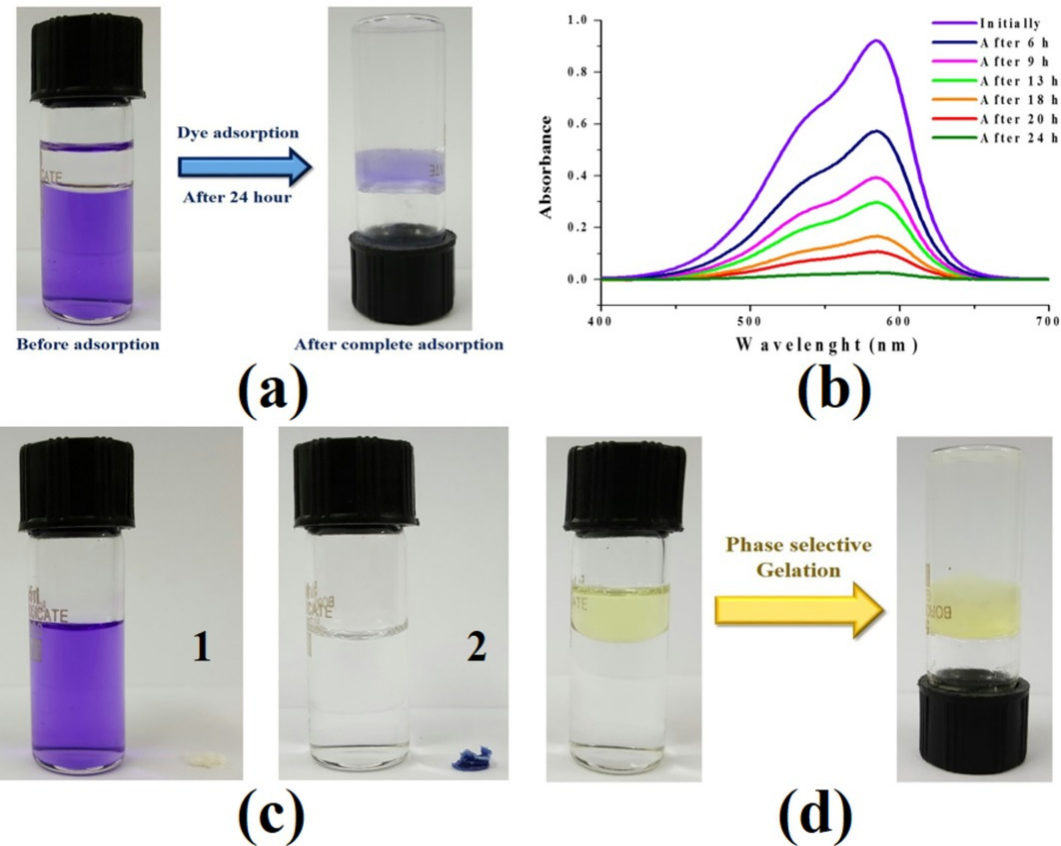
(**a**) Phase selective gelation (toluene/water) followed by absorption of dye from the aqueous layer; (**b**) UV-vis spectra of dye absorption over time; (**c**) Aqueous methyl violate solution (0.02 mM) (left) and the solution after the addition of a xerogel made in toluene (right); (**d**) Phase selective gelation of a diesel-water mixture. Reprinted with permission from [61]. Copyright 2017 John Wiley & Sons, Inc. (Hoboken, NJ, USA).

The gelator **27b** in Figure 19 also showed applications in environmental remediation. Phase-selective gelation was found in several mixtures of organic solvents and water. In Figure 75a, biphasic solutions of methyl violate solution and toluene were heated with the gelator and the toluene layer formed a gel. After 24 h, the aqueous layer appears clear and most of the methyl violate had diffused into the organogel. UV-vis spectrum shown in Figure 75b shows the near complete absorption of the dye into the organogel. Figure 75 also shows that a xerogel was capable of removing methyl violet from the aqueous phase effectively [61]. The last image in Figure 75 (**d**) shows the phase selective gelation of diesel fuel, indicating this molecule may be useful in applications of fuel spill cleanup.

### 4.2. Drug Delivery

Drug delivery refers to the technology utilized to present a drug to a desired active site in the body for drug release and absorption, or the subsequent transport of the active ingredients across biological membranes to the site of action. Carbohydrates are biocompatible, relatively inexpensive, structurally easy to functionalize, and overall green. There is an enormous amount of studies on gels for use as drug delivery systems and many of these examples use oligomers or polymers made from scaffolds of carbohydrates [133,134]. Many of these hydrogels are porous and can swell and shrink, which allows for them to be exemplary models for drug delivery applications. Whilst this review mainly focuses on gelators made from small molecules, it is deemed pertinent to mention a few examples of polymers that have been successful in the field of drug delivery. The next section will showcase several examples utilizing carbohydrate-based gels for drug delivery applications.

Protein delivery systems, which allow proteins to retain their integrity and biological activity, are one of the key components in the development of therapeutic proteins and peptides. Xu et al. have successfully synthesized a biocompatible and biodegradable hydrogel based on a naturally occurring polysaccharide which has been evaluated as a carrier for protein drugs [135]. The epichlorohydrin crosslinked hydroxypropyl pachyman hydrogel exhibited significant pH-sensitivity, which was favorable for protein release in a simulated intestinal medium. The self-assembly has been found to encapsulate up to 97.6 wt% of various protein drugs [135]. Two model protein drugs, bovine serum albumin and lysozyme, were studied using the hydrogel. It was noted that the protein stability was not affected during encapsulation and release. Moreover, the insulin-loaded hydrogel was effective in reducing blood glucose level in diabetic animal models and showed no evidence of cytotoxicity in vitro or in vivo. The synthesized hydrogel shows favorable features as a promising delivery carrier candidate for targeted delivery of protein drugs to specific sites [135]. Another example using a polysaccharide-based hydrogel for drug delivery was reported by Yoo et al. [136]. They utilized a glycol chitosan scaffold that was covalently attached to glycidyl methacrylate which was later mixed with doxorubicin hydrochloride [136]. After the mixture was exposed to visible blue light (430–485 nm), a hydrogel was formed with the doxorubicin hydrochloride entrapped within its matrix. Overall, there was an initial surge in the release of the drug during the first 18 h, but later a slow, sustained release was recorded over 7 days. In addition to that, the researchers found that the drug loaded hydrogel had a better anti-tumor effect on thyroid cancer as opposed to the drug on its own [136]. This shows the hydrogel has great potential for drug delivery systems.

**Scheme 2:**
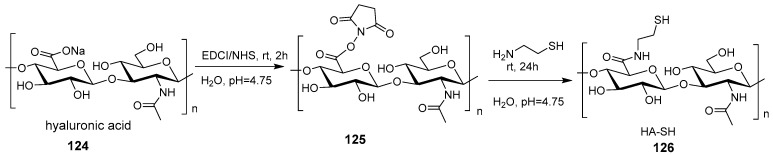
Synthesis of a thiolated hyaluronic acid copolymer for drug delivery, EDCI: 1-ethyl-3-(3-dimethyllaminopropyl)carbodiimide hydrochloride, NHS: N-hydroxysuccinimide [137], n represents the number of repeating units.

He et al. reported a novel injectable hydrogel prepared from hyaluronic acid (HA) with a thiol group attached as shown in Scheme 2 [137]. Hydrogel formation occurred after the free thiol groups in HA-SH **126** were oxidized to give cross-linked disulfide bonds [137]. They studied the release of doxorubicin, metformin and sorafenib from the hydrogel of compound **126**, in the presence of different concentrations of the redox reagent dithiothreitol (DTT). The hydrogels loaded with the drugs were injectable and remained stable at physiological conditions with a swift and precise release of the drugs in the presence of DTT. In addition to that, the hydrogel was able to encapsulate multiple drugs at the same time and the in vitro as well as in vivo assays demonstrated synergetic effect against tumor cells [137]. The results have shown that localized hydrogels loaded with multiple drugs have improved efficacy while reducing systemic toxicity.

Kannan et al. researched the use of a carbohydrate-based low molecular weight gelator **30a** (Figure 21) as a drug carrier for a local anesthetic [64]. Compound **30a** was found to be capable of entrapping hydrophilic dyes and prilocaine hydrochloride, a local anesthetic drug. In vitro drug release studies found an inverse relationship between gelator concentration and diffusion rate. The prilocaine HCl release profile aligns with the Peppas–Korsmeyer model, which depicts a non-Fickian diffusion mechanism associated with diffusion from these supramolecular gels [64]. In vitro cytotoxicity studies showed that these gels have high biocompatibility suggesting these poly aryl ether glucose cored dendron derivatives are promising candidates for localized and sustained drug release for topical applications.

**Figure 76:**
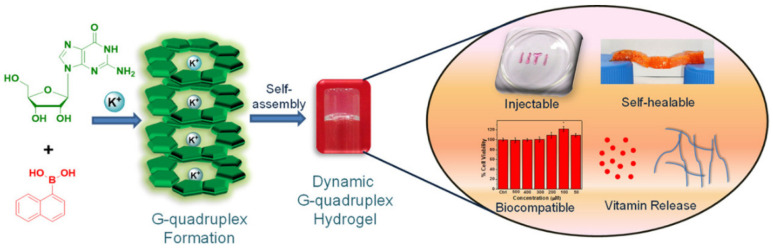
G-Quadruplex formation of a cytidine-based gelator and its features. Reprinted with permission from [138]. Copyright 2020 American Chemical Society.

Guanosine derived gelators, have also demonstrated their capability as sustained delivery systems. A very recent publication outlined that the G-quadruplex hydrogel formed with potassium in Scheme 1, is in fact biocompatible with self-healing properties and can be utilized for the sustained release of vitamins [138]. A diagram summarizing its self-assembly and properties can be seen in Figure 76. Release studies were performed with vitamin B_2_ and B_12_ separately, both were released gradually without interference from other factors. However, in the presence of an acidic environment vitamin release was much faster than at physiological pH [138]. Additionally, the release profile of doxorubicin was measured in an acidic environment from the hydrogel; the results showed that approximately 76% of the entrapped cancer drug was released in just 15 h showing its potential to be used in pH responsive drug delivery systems [138]. The researchers credited the hydrogel’s stimuli-responsiveness to the presence of the boronate ester.

### 4.3. Biomedical Applications: Antibacterial Agents and Wound Healing

Carbohydrate-based materials have also been studied as antibacterial agents that can prevent biofilm formation. A few gluconamide-based amphiphiles depicted in Figure 23 have been recently reported in the literature for these specific applications [68]. The antibacterial studies were carried out using both compound **32a** and **32b** for several bacterial strains including *Listeria monocytogenes* (LM), *Staphylococcus aureus* (SA), *Solmonella enterica Typhimurium* (STm) and *Pseudomonas aeruginosa* (PA) [68]. The in vitro studies demonstrated that the abovementioned compounds were able to inhibit bacterial growth by a large percentage. Compound **32a** was able to inhibit the growth of PA, SA, STm, and PA at a concentration of 400 µg mL^−1^ by about 70%. On the other hand, glycolipid **32b**, a combination of lipids with different chain lengths, showed better antibacterial activity and was typically effective at 200 or 400 µg mL^−1^ for the same bacteria [68]. Consequently, further studies also confirmed that both gelators were able to inhibit the growth of biofilm within the species mentioned above [68]. Overall, the gluconamide gelators presented have the potential to be utilized in several applications where bacterial film formation needs to be minimized such as in wound healing, implanted medical devices and chronic sinusitis [68].

**Figure 77:**
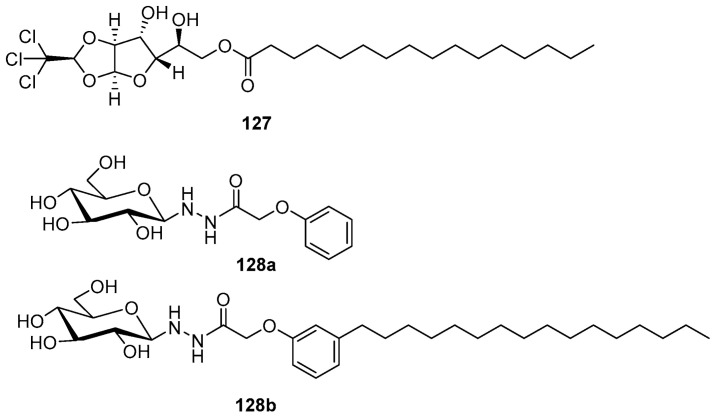
Gelators for antibacterial and wound healing applications [139,140,141].

Several other glycolipids which can function as gelators were also reported with wound healing and antibacterial properties. A few examples are shown in Figure 77 [139,140]. The glycolipid **127**, synthesized from α-chloralose using Novozyme 435, was able to form a gel in DMSO:H_2_O (*v*/*v* 1:4) at 2.5% *w*/*v*, eucalyptus oil at 1% *w*/*v* and an oleogel formed in paraffin oil also at 1% *w*/*v* [139,140]. The oleogel was utilized to encapsulate curcumin and the morphological studies showed fibrous networks throughout the gel matrix [139]. In addition to that, they exhibited thixotropic properties, with injectable and self-healing features that would allow for their utilization in vivo [139,140]. Solutions of compound **127** were investigated for their ability to inhibit bacterial growth of *Pseudomonas aeruginosa* (PA), Methicillin-resistant *Staphylococcus aureus* (MRSA) and Methicillin-susceptible *Staphylococcus aureus* (MSSA). These studies revealed that compound **127** in its natural state was able to inhibit the growth of MSSA and MRSA, but had a smaller effect on PA [139]. The molecule was shown to be more successful at hindering biofilm formation for all bacterial strains [139]. Both the composite gel with the curcumin entrapped as well as the oleogel, were able to assist with wound closure in diabetic induced Wistar rats [139,140]. This was achieved through the gels enhancing collagen synthesis and regulating cell proliferation and overlapping phases of inflammation, as well as controlled the production of free radicals from the wound [139,140].

Compounds **128a–b**, which were also described as antibacterial agents, were efficient gelators for several oils and other hydrophobic solvents. However, when tested in water, they formed foam which was stable at room temperature for approximately 6 h [141]. Solutions of compound **128a–b** were utilized to investigate their ability to disrupt biofilm formation. Derivative **128a** was more effective at interrupting the growth of biofilms from uropathogenic *E. coli* and *Salmonella enterica* Typhimurium, whilst **128b** was able to disassemble biofilms formed by *Staphylococcus aureus* and *Listeria monocytogenes*, which are Gram positive [141]. The authors believe that these compounds can be applied as cleaning agents either as a solution or in the gel form.

Cytidine derivatives have also been evaluated in the literature for their potential usage as antibacterial agents and for wound healing purposes. Two examples of this type of gelator formed hydrogels by combining silver nitrate, silver acetate, or boric acid, with cytidine [142,143]. The resulting hydrogels had inherent antibacterial activity from the silver ions, which are well-known antibiotics [142,143]. The hydrogel with silver nitrate was able to inhibit the growth of *Escherichia coli, Staphylococcus aureus* and *Pseudomonas aeruginosa* and growth decreased as the volume of the hydrogel increased [142]. In addition, the hydrogel was also found to boost burn wound closure in ICR mouse models by enhancing the growth of cutaneous tissue [142]. These properties, along with the thixotropic characteristics of the gel, allow for it to have great potential for utilization in tissue engineering and other biomedical applications. On the other hand, the hydrogel made from the addition of silver acetate was not very effective against *Streptococcus pneumoniae* and *Staphylococcus aureus,* but was more potent against a multidrug resistant strain of *Morganella morganii*, *Pseudomonas aeruginosa*, *Klebsiella pneumoniae* and *Escherichia coli*. Interestingly, the hydrogel that was made with silver acetate did not discolor the tissues at the application site, leading the authors to suggest that this is advantageous over using silver nitrate in antibacterial gels [143].

### 4.4. Tissue Engineering

Supramolecular gels are particularly important in the biomedical field due to their ability to mimic cellular environments. In culturing stems cells, the stiffness of supramolecular gels has been observed to direct cell differentiation [144,145]. Tissue engineering or regenerative medicine is a rapidly evolving field that has approximately 30 years’ worth of research so far. It is a very appealing area of research that combines many disciplines with the aim of restoring human tissue to cure diseases and help with organ failure [146,147,148]. The main factors that are to be considered when designing scaffolds for tissue engineering are; the material must be biocompatible, biodegradable, possess a mechanically strong porous network for structural support and the movement of nutrients and wastes, contain biochemical cues or surface chemistry for the attachment of cells, promote proliferation and differentiation of cells as well as the substances produced during the degradation process must be non-toxic [146,149,150,151]. Throughout the years, many materials, such as polymers, have been designed for this purpose and research is still ongoing. However, many supramolecular biomaterials such as gels have been studied for this purpose and many show promising results. Herein the preparation of gels from carbohydrates for applications in tissue engineering will be reviewed.

**Figure 78:**
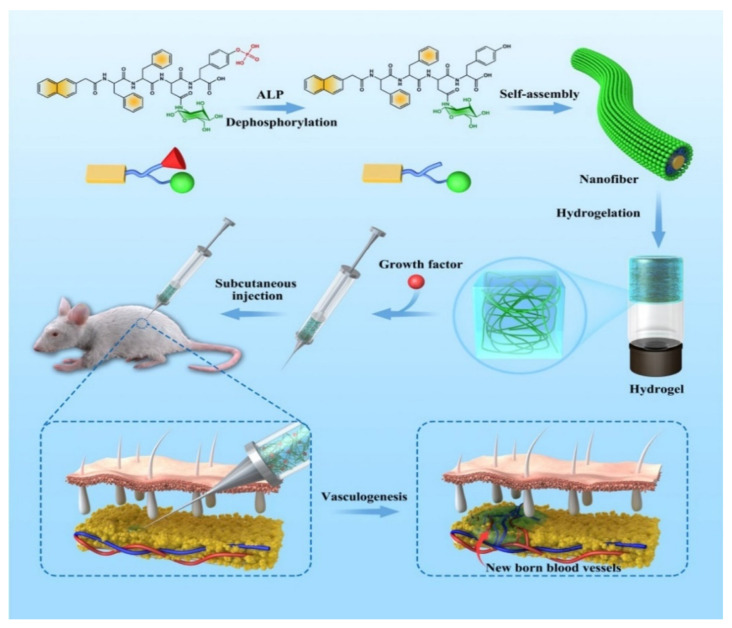
Schematic illustration of the glycopeptide self-assembly and its utilization as a cell growth medium. Reprinted with permission from [152]. Copyright 2020 American Chemical Society.

Although many carbohydrate based LMWGs have not been studied for applications in tissue engineering, there are a few references in the literature that will be reviewed here. The first example is a glycopeptide hydrogel and the other is an alkyl galactonamide gelator [65,152]. The researchers created a novel glycopeptide that could be cleaved by alkaline phosphatase (ALP) after which gelation would occur [152]. The molecule was comprised of a peptide segment (Phe-Phe-Asp-Tyr(H_2_PO_3_), a naphthyl group and D-glucosamine as the sugar component [152]. Each of these moieties had an important function for the desired application of the gelator. The peptide segment containing the Tyr(H_2_PO_3_) serves as the substrate for enzymatic catalysis which then allows for rapid self-assembly [152]. Self-assembly of the compound is controlled by the naphthyl moiety and the Phe-Phe dipeptide by way of increased π-π interactions arising from the aromatic rings. Consequently, the D-glucosamine unit is there to stimulate cell growth and attachment [152]. The schematic diagram in Figure 78 shows the mechanism of self-assembly and usage of the hydrogel in tissue engineering.

At a concentration of 0.6 wt % and pH of 7.4, the glycopeptide does not form a gel in water, but when treated with ALP at a concentration of 10 units/mL it forms a gel [152]. The gel had the typical fibrous morphology that is generally seen as evidenced by their transmission electron microscopy (TEM) images. The scanning electron micrograph images also illustrated a porous 3D structure formed by the gels [152]. The rheological data confirmed the viscoelastic properties of the gel as the storage modulus (G′) was greater than the loss modulus (G”). The gel was then investigated for its ability to act as a cell culturing matrix for the growth of human umbilical vein endothelial cells (HUVECs). The cells were able to adhere to the gel and proliferate successfully [152]. This and other studies that were carried out demonstrated that the gel was very biocompatible. The growth of the cells was attributed to the glucose moieties, which allowed for successful adhesion of the cells to the gel via sugar-receptor interactions [152]. Other rheological data confirmed the sheer-thinning and recovery properties of the gel, which allowed for it to be employed as an injectable hydrogel [152]. This property was then utilized for in vivo applications where the gel was loaded with deferoxamine (DFO) which is a drug that promotes vascularization and then a subcutaneous injection allowed for it to be embedded on the dorsal side of the mice [152]. This was evaluated for several days and they found that even though the volume of the gel steadily decreased over time, the gel matrix was able to support the development of blood capillaries within the mice [152]. Overall, the glucose incorporated hydrogel was able to be utilized as an extracellular matrix for tissue engineering.

In a second article, a gelator molecule created from *N*-heptyl-galactonamide (**31b** in Figure 22), was synthesized and utilized as a material for growing adult human neuronal stem cells (hNSCs) in 3D [65]. The molecule was able to form a hydrogel at 1 wt % and the morphology was examined under TEM and SEM which both illustrated a fibrous morphology [65]. Upon testing the ability of the gel to grow cells, there were no additional growth factors or adhesion molecules within the gel matrix [65]. Using the technology of a confocal microscope, the researchers were able to observe the neuronal cells infiltrating deep (~200 μm) into the gel network to generate neurosphere-like structures [65]. The cells were able to form a rather compact neurofilament networks after only 7 days of culture. They were also able to differentiate into glial and neuronal cells by the generation of neurite outgrowths along the gel fibers [65]. This simple gelator molecule definitely exhibited all the innate properties that biomaterials must have to be employed in regenerative medicine for the growth of neuronal cells. This gelator is advantageous for this application because preparation includes a simple one-step reaction that can be obtained and the gram scale and the addition of several other growth factors may not be trivial [65].

### 4.5. Shaping, Printing, Self-Healing and Thixotropy

Shaping gels formed from LMWGs has proven to be a very difficult task. Supramolecular gels formed by small molecules are typically fragile and thus difficult to manipulate without destroying the gels. The easiest way to shape a gel is to form the gel in a mold, which is the typical procedure in the discovery of LMWGs, as the typical testing involves adding a small amount of gelator to a vial, adding solvent, and then applying a stimulus to trigger gelation. Rheological properties of gel materials can be adjusted through controlling the gelator concentrations and through annealing processes [144,153]. Some peptide-based LMWGs have been shown to be capable of withstanding the extrusion process for 3D printing, such as the LMWGs reported by Adams et al. [154]. There are fewer examples of carbohydrate based LMWGs for 3D printing, but many more successful results have been reported using polysaccharides and their derivatives for 3D printing and tissue engineering [155,156]. In order for supramolecular gels made from LMWGs to be capable of 3D printing, the gels must possess thixotropic properties. Thixotropy is a shear thinning property in which a material becomes less viscous when a shear force is applied, followed by a time-dependent return to a more viscous state. In extrusion-based 3D printing, in order for the formed gel to be extruded through the nozzle of the 3D printer, the gel must undergo shear thinning first, and then once extruded, the material must become more viscous and return to a stable gel [154].

**Figure 79:**
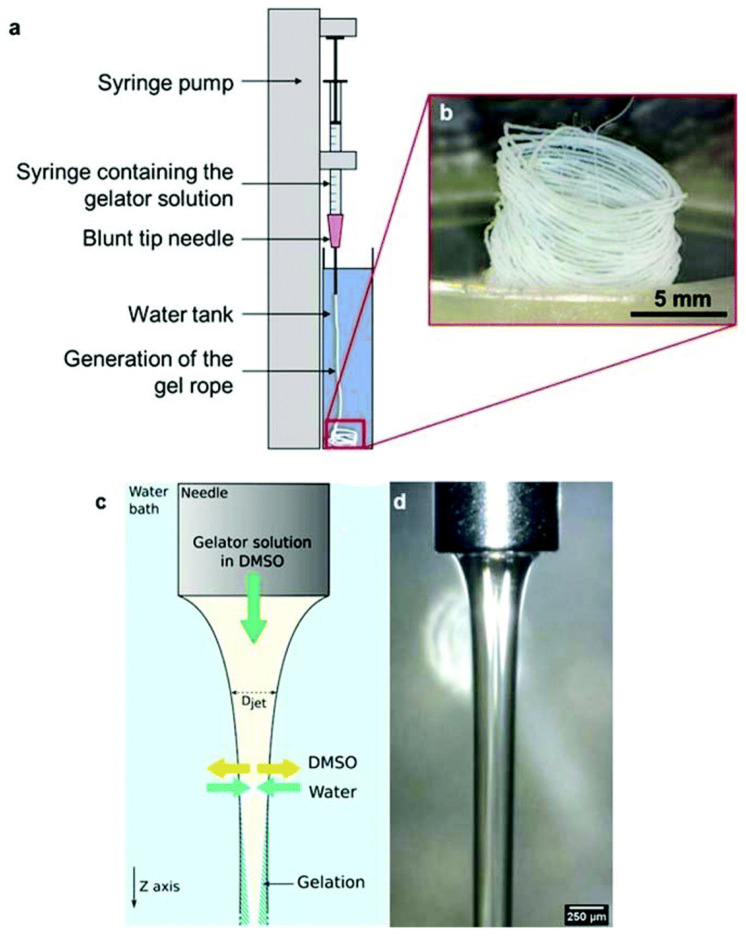
(**a**) Apparatus setup for wet spinning gel. (**b**) Magnification of the coiled gel filament after extrusion, gelator solution: 4 wt % **31b** solution in DMSO (50 μL min^−1^, 20 G needle, ID 600 μm. (**c**) Schematic explanation of the wet spinning method. (**d**) Image of the DMSO jet after escaping the needle at a speed of 30 μL min^−1^. Reprinted with permission from [66]. Copyright 2019 Royal Society of Chemistry under the creative commons license.

An alternative to extrusion-based printing is to use rapid solvent exchange to form gels in situ. In this technique, a gelator is dissolved in a solvent and the gelator solution is injected into a bath holding a solvent in which the gelator is insoluble. A rapid solvent exchange occurs causing rapid self-assembly and the formation of a gel filament or print [66]. Fitremann et al. showed that well-organized hydrogel filaments could be formed through DMSO-H_2_O solvent exchange [66]. The hydrogels formed by N-heptyl-D-galactonamide (**31b** in Figure 22) cannot be successfully extruded because they are fragile and undergo syneresis when they undergo a shear force, similar to many other supramolecular gels formed from LMWGs. In this study, a DMSO solution of N-heptyl-D-galactonamide was injected into a water bath at an optimized rate controlled by a syringe pump, resulting in the formation of a continuous uniform hydrogel filament shown in Figure 79. This result opens up the possibility of 3D printing non-thixotropic gels, as the gel can be formed on a surface through this solvent exchange.

In a later study, Fitremann et.al. showed that solvent exchange can be used to 3D print this non-thixotropic carbohydrate-based supramolecular gel [67]. Using a syringe pump, XY-translation stage and a handheld Z-adjustable platform, a DMSO solution of N-heptyl-D-galactonamide (**31b** in Figure 22) was injected into a water bath to print a gel onto a glass slide covered in a hydrophobic polycarbonate membrane. The flow rate was adjusted until good adhesion to the polycarbonate membrane was achieved. Using this technique, 3D printed biocompatible supramolecular gel scaffolds can be custom printed using carbohydrate-based LMWGs. This work opens the door for 3D printing other non-thixotropic supramolecular gelators.

The methyl glucoside derivative **14b** (Figure 7) formed a translucent silicone oil gel that is capable of extrusion. Rheological measurements were used to evaluate the strength of the extruded gel. Frequency sweep curves show a storage modulus (G′) that is consistently larger than the loss modulus (G″), indicating the extruded soft material retained the viscoelastic behavior of a gel. The thixotropic properties of the gel produced from compound **14b** were also investigated in this study. Gels of **14b** in ethanol/water 1:1 and squalene were sheared using a vortex mixer and the gels were found to reform within 1 h sitting at room temperature undisturbed [47].

**Figure 80:**
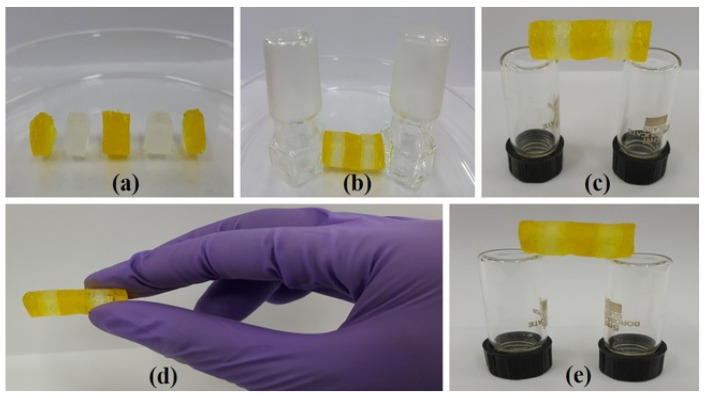
Mesitylene gels at 0.3% *w*/*v*. (**a**) Pieces of doped and undoped gel in alternating order; (**b**) Compressing the pieces to promote self-healing; (**c**) Rod of gel after self-healing; (**d**) Handheld gel rod; (**e**) Image showing the diffusion of the azo dye into the undoped segments of the gel rod. Reprinted with permission from [61]. Copyright 2017 John Wiley & Sons, Inc.

Along with thixotropy, self-healing properties are very important in shaping supramolecular gels. The mannose-based low molecular weight organogelator **26** in Figure 19 exhibited significant self-healing capabilities, as well as phase-selective aqueous-organic gelation [61]. Figure 80 shows two blocks of gels, one of which was doped with 4,4′-dihydroxyazobenzene, that were cut into pieces, stacked in alternating order, and pressed together to form one solid bar, demonstrating significant self-healing capability. Diffusion of the azo dye indicates diffusion across the fusion interface [61]. Rheological studies were carried out to assess the reversibility and thixotropic properties of the organogels. A 0.3% mesitylene gel was subjected to an initial constant strain of 0.1%, followed by increasing the strain to 210% for a period and then returning the strain to 0.1%. Several cycles of increasing and returning the strain were carried out [61]. Initially, the storage modulus (G′) was higher than the loss modulus (G”), indicating the viscoelastic behavior of a gel. After the strain was increased, the loss module (G″) was higher than the storage modulus (G′), indicating the break-down of the gel to a more solution-like nature. After reducing the strain, the gel regained its mechanical strength indicated by the storage modulus (G′) becoming higher than the loss modulus (G″). After each cycle, the gel regained up to 90% of its mechanical strength [61].

Chlorobenzene gels made from compound **34c** in Figure 25 with capric acid, myristic acid and stearic acid were found to possess incredible thixotropic properties [70]. Upon destruction of the gel through mechanical forces, instantaneous self-healing occurred within seconds. The self-healing ability of these gels was further studied by cutting cylinders of gel into pieces and stacking them back together. Interestingly, pieces of gel made from different gelators were found to be capable of fusing together. A chlorobenzene gel made from **34c** and stearic acid was found to be highly transparent, which makes it useful in flexible optical devices [70]. This gel was also used to prepare a gel film and in an extrusion study. Additionally, since the **34c** and stearic acid combination spontaneously gelled aromatic solvents, a phase selective gelation, along with a dye removal study was carried out. Powdered **34c** and stearic acid were added to a biphasic mixture of o-xylene, toluene, ethylbenzene, 1,3,5-trimethyl-benzene, benzene, and water. Within 15 min, all of the aromatic solvents were solidified. When inverted, the gels were strong enough to withhold the weight of the aqueous layer. Additional studies in phase selective gelation showed that these aromatic solvents can be solidified, removed from the aqueous phase and distillation could be used to recover the gelator and aromatic solvents [70]. In an additional study, **34c** and stearic acid powder was added to a biphasic solution of benzene and water contaminated with methylene blue. Within 5 h, the gel that was formed had adsorbed nearly all the methylene blue from the aqueous phase. These novel multi-functional two-component gel systems have many interesting properties and are potential candidates in various applications.

## 5. Conclusions

We have highlighted the recent discoveries of carbohydrate-based organogelators and hydrogelators as well as their versatile applications in many different areas. This review mainly focused on publications from January 2017 to December 2020 and several other papers that were published earlier. Carbohydrates and other naturally occurring starting materials have been extensively studied for generating soft materials because they are readily available and biocompatible. The naturally occurring common D-monosaccharides that have been studied for the design of the LMWGs included in this review are glucose, glucosamine, mannose, galactose, arabinose and ribose. The C-1 reduced and oxidized sugar derivatives including alditols and glyconic acid-derived molecules were also discussed. The majority of the sugars were functionalized by the formation of acetals or esters as well as the introduction of hydrophobic components in the molecule. Since gelation requires a balance of intermolecular interactions, we hope the examples reviewed here can shed light on how the modification of monosaccharides can lead to desired properties. LMWGs have been utilized for diverse fields such as environmental remediation, biomedical applications, as new materials for tissue engineering and in many other areas. Incorporating various functional groups to enable the design of multiple stimuli-responsive gelation systems has also been an important subject of study, which results in a wider scope of applications. The stimuli-responsive gelators that are sensitive to acids, bases, enzymes, photo radiation and ions were also discussed. Other applications, such as using sugar-based gelators for 3-D printing, catalysis, tissue engineering and drug delivery, are fascinating, and will likely produce new knowledge on both basic science and advanced technology. The design of gelators with predictable properties is still challenging; however, the structure-based design from certain templates that we have shown in this review can be utilized for future studies. Using renewable resources for the creation of functional soft materials has significance in various research fields and is economically valuable. The continuous pursuit to develop novel gel-based soft materials is a growing research endeavor which will require collaborations from experts in multiple fields.
